# Immunologic and pathologic characterization of a novel swine biomedical research model for eosinophilic esophagitis

**DOI:** 10.3389/falgy.2022.1029184

**Published:** 2022-11-14

**Authors:** Lizette M. Cortes, David Brodsky, Celine Chen, Tiffany Pridgen, Jack Odle, Douglas B. Snider, Glenn Cruse, Arina Putikova, Mia Y. Masuda, Alfred D. Doyle, Benjamin L. Wright, Harry D. Dawson, Anthony Blikslager, Evan S. Dellon, Scott M. Laster, Tobias Käser

**Affiliations:** ^1^ Department of Population Health and Pathobiology, College of Veterinary Medicine, North Carolina State University, Raleigh, NC, United States; ^2^ Center for Food Allergy Modeling in Pigs (CFAMP), Comparative Medicine Institute, North Carolina State University, Raleigh, NC, United States; ^3^ Department of Biological Sciences, North Carolina State University, Raleigh, NC, United States; ^4^ USDA, ARS, Diet, Genomics and Immunology Laboratory, Beltsville, MD, United States; ^5^ Department of Clinical Sciences, North Carolina State University, Raleigh, NC, United States; ^6^ Laboratory of Developmental Nutrition, Department of Animal Science, North Carolina State University, Raleigh, NC, United States; ^7^ Department of Molecular Biomedical Sciences, North Carolina State University, Raleigh, NC, United States; ^8^ Division of Allergy, Asthma, and Clinical Immunology, Department of Medicine, Mayo Clinic Arizona, Scottsdale, AZ, United States; ^9^ Department of Immunology, Mayo Clinic, Rochester, MN, United States; ^10^ Department of Immunology, Mayo Clinic Arizona, Scottsdale, AZ, United States; ^11^ Section of Allergy and Immunology, Division of Pulmonology, Phoenix Children’s Hospital, Phoenix, AZ, United States; ^12^ Division of Gastroenterology and Hepatology, Department of Medicine, Center for Esophageal Diseases and Swallowing, University of North Carolina School of Medicine, Chapel Hill, NC, United States

**Keywords:** pig, food allergy, animal model, immunology, gastrointestinal, eosinophilic esophagitis

## Abstract

Eosinophilic esophagitis (EoE) is a chronic allergy-mediated condition with an increasing incidence in both children and adults. Despite EoE's strong impact on human health and welfare, there is a large unmet need for treatments with only one recently FDA-approved medication for EoE. The goal of this study was to establish swine as a relevant large animal model for translational biomedical research in EoE with the potential to facilitate development of therapeutics. We recently showed that after intraperitoneal sensitization and oral challenge with the food allergen hen egg white protein (HEWP), swine develop esophageal eosinophilia—a hallmark of human EoE. Herein, we used a similar sensitization and challenge treatment and evaluated immunological and pathological markers associated with human EoE. Our data demonstrate that the incorporated sensitization and challenge treatment induces (i) a systemic T-helper 2 and IgE response, (ii) a local expression of eotaxin-1 and other allergy-related immune markers, (iii) esophageal eosinophilia (>15 eosinophils/0.24 mm^2^), and (iv) esophageal endoscopic findings including linear furrows and white exudates. Thereby, we demonstrate that our sensitization and oral challenge protocol not only induces the underlying immune markers but also the micro- and macro-pathological hallmarks of human EoE. This swine model for EoE represents a novel relevant large animal model that can drive translational biomedical research to develop urgently needed treatment strategies for EoE.

## Introduction

Food allergies (FAs) are defined as antigen (Ag)-specific immune responses against food proteins (1). FAs are endemic in our society with recent surveys suggesting approximately 8% of children (2) and 11% of adults (3) display FAs. FAs to peanut, milk, and egg are among the most common although over a hundred food allergens have been identified worldwide (4). Foods that provoke FA also vary among geographic and demographic populations indicating that genetic and environmental factors are also important for expression of FAs (5). The symptoms of FAs include angioedema of the lips and mouth, urticaria, diarrhea, emesis, respiratory symptoms, and anaphylactic responses (6). Certain FAs are expressed primarily in children and will wane as the child matures; however, allergies can also persist into adulthood. In other individuals FAs will begin in adulthood and remain lifelong, disabling disorders accompanied by significant pathology (7).

Due to intensive study of a number of highly developed mouse models of FAs (8), the underlying immune cellular and molecular mechanisms of FA are relatively well understood. Food allergies are initiated by antigen processing and presenting cells (APCs): they take up the food protein, process it, and present the resulting peptides to T cells. While a healthy response to food proteins drives a Treg-response, FA patients develop a T-helper 2 (Th2) immune response (9). These Th2 cells secrete IL-4 and IL13; both can drive antibody production towards the isotype IgE. Innate immune cells like mast cells and basophils have IgE receptors and bind the allergen-specific IgE leading to their sensitization. Once sensitized, these cells can degranulate and induce food allergic symptoms (10). Why certain humans respond in this manner is not fully understood; yet, it is likely due to a number of factors including low levels of vitamin D (11), reduced microbial exposure (12), alterations in the microbiome (13), skin Ag exposure (14), and exposure to advanced glycation end-products (15). In addition, not all FAs use the same mechanism; there are three classes of allergic disorders—(i) classic, IgE-FA dependent, (ii) mixed IgE/non-IgE, and (iii) non-IgE-dependent disorders like EoE. IgE-dependent effector responses are seen in the majority of food allergic patients, with physiological symptoms arising from the release of mast cell and basophil mediators (16). Mixed IgE/non-IgE FAs are less understood, involve both IgE and cellular immune responses (17, 18), and their symptoms often arise delayed with the potential of chronic disease (19). Non-IgE-dependent, T cell-mediated disorders of the GI tract also have been noted (20) including an inflammatory disorder of the esophagus known as eosinophilic esophagitis (EoE) (21). In this disorder, an allergen may directly enter the esophageal tissue, perhaps through a disrupted esophageal mucosal barrier, leading to an abnormal stimulation of the CD4 T cell subset T-helper 2 (Th2). While the systemic Th2 response can lead to eosinophil activation and proliferation in the bone marrow, local esophageal Th2 cells can induce the production of the eosinophil-attracting chemokine eotaxin. While the precise role of esophageal eosinophils in EoE remains unclear, they can produce a range of inflammatory and cytotoxic substances that may contribute to tissue damage and fibrosis (22, 23). These pathological changes can then cause the EoE-associated clinical symptoms like dysphagia, reflux, vomiting, and epigastric pain that negatively impact the health-related quality of life in both youth (24) and adults (25). Left unchecked, eosinophilic esophageal inflammation in humans can progress to fibrosis, manifested by esophageal remodeling, strictures, and food impactions (26–28).

While mouse models have greatly contributed to the understanding of the immune mechanisms underlying EoE, they also have limitations in regard to translatability. These limitations are mainly based on differences between mice and humans regarding anatomical size, esophageal physiology, and lifespan (29, 30). These limitations can hinder the development of both prophylactic or therapeutic treatments like small molecules, monoclonal antibodies (31–36), or oral, sublingual, and epicutaneous immunotherapies (1). Selecting among these and future treatments for testing in human clinical trials could be facilitated by a translational large animal model like swine. Swine have high immunological and physiological similarities to humans and represent an important biomedical animal model [reviewed in (37, 38)]. Importantly for esophageal diseases, the anatomical size of the esophagus permits assessment of pathology in a similar fashion to human subjects—for example, endoscopy can conveniently be performed on swine. Swine also share a relevant feature that is absent in rodents—esophageal submucosal glands (ESMGs). These ESMGs are crucially involved in esophageal repair (39–41). Based on the long lifespan of swine, long-term studies into chronic diseases such as EoE are also feasible in swine. For these reasons, we have been studying the food allergic response in swine. In previous studies, the Wilkie group successfully induced food allergy in swine using the hen egg white protein (HEWP) ovomucoid (42, 43). Based on these studies, we chose HEWP as allergen and have shown previously that swine can indeed develop a FA-like and EoE-like response to the HEWP component ovalbumin [OVA, (33)]. In that study, animals sensitized by injection of HEWP with cholera toxin and followed by an oral gavage of HEWP developed gastrointestinal distress, eosinophilic infiltration of the esophagus, skin rashes, and OVA-specific T cells. Herein, we used a more physiological allergen protein challenge by daily feedings of HEWP for 1 week. Furthermore, we include a more in-depth analysis of the systemic and local immune responses as well as allergen-induced histologic and gross pathologic changes. Thereby, we demonstrate that our HEWP sensitization and challenge protocol not only induces systemic and local immune responses seen in human EoE patients (e.g., a Th2 response, anti-OVA IgE, and esophageal eotaxin production), but also the micro- and macro-pathological changes associated with human EoE (e.g., esophageal eosinophilia on histologic exam, and white exudates and linear furrows on endoscopic exam). In summary, we demonstrate that swine can be used as a valuable large biomedical animal model for translational research in EoE.

## Materials and methods

### Study design: *In vivo* sensitization and oral challenge setup

**Figure 1:**
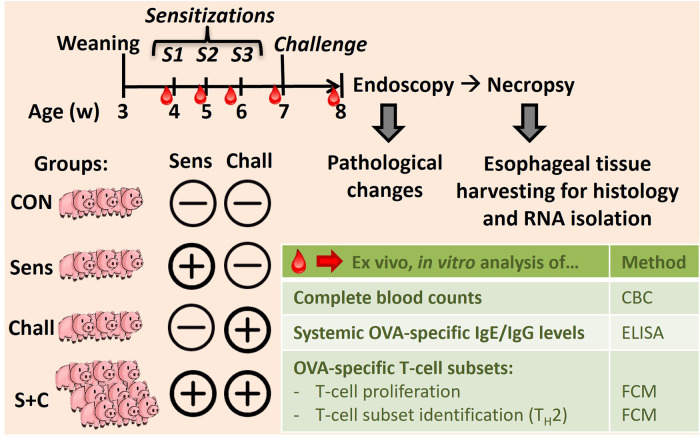
In vivo animal trial setup to induce eosinophilic esophagitis (EoE) in swine. Eighteen 4-week-old weaned swine were blocked and randomly divided into four groups: (i) the control group (CON, *n* = 3), (ii) the sensitization group (Sens, *n* = 3), (iii) the challenge group (Chall, *n* = 3), and (iv) the sensitization plus challenge group (S + C, *n* = 9). Sensitization consisted of three weekly intraperitoneal injections (Cholera toxin + hen egg white protein, HEWP). One week after the last sensitization, swine were challenged orally with HEWP daily for 1 week. Blood was collected weekly for complete blood counting, and the isolation of serum and peripheral blood mononuclear cells (PBMC). At the end of the study, swine were endoscoped, euthanized, and esophagi were collected for tissue harvesting.

As shown in Figure 1, eighteen 4-week-old weaned piglets raised without exposure to HEWP were included in the study. The swine were blocked by sex and weight and then randomly assigned into one of four treatment groups. Based on previous data demonstrating that swine develop signs of EoE only after both sensitization and challenge (33), swine were distributed asymmetrically: (1) non-sensitized/non-challenged (CON, *n* = 3), (2) HEWP-sensitized/non-challenged (Sens, *n* = 3), (3) non-sensitized/HEWP-challenged (Chall, *n* = 3), and (4) HEWP-sensitized/HEWP-challenged (S + C, *n* = 9). The swine were fed HEWP-free feed and monitored two times/day in accordance with the USDA recommendations. Sensitization of swine in the HEWP-sensitized groups was performed intraperitoneally with 500 μg HEWP and 10 μg cholera toxin at 0, 7, and 14 days post first sensitization (dps). From 21 to 28 dps, swine in the HEWP-challenged groups were fed by hand a daily dose of 10 g of HEWP mixed into commercial maple syrup. Non-challenged swine received commercial maple syrup without HEWP. Starting at 0 dps, blood samples were collected weekly. After the completion of the week-long daily oral challenges, an upper endoscopy was performed to investigate signs of EoE and to take mucosal biopsies from the proximal, mid, and distal sections of the esophagus. After the endoscopy, the swine were sacrificed by lethal injection of euthasol (390 mg/ml pentobarbital, 50 mg/ml phenytoin, Virbac AH, Inc, Carros, France). Then, the esophagus was resected *en bloc*, opened and tissue was collected for various downstream analyses—histology, immunohistochemistry, and RNA expression. Of note, one swine in the HEWP-sensitized/HEWP-challenged group died after the first oral challenge. While no overt cause of death was found, the most significant finding on necropsy was a mild eosinophilic enterotyphlocolitis. The experimental procedures were approved by the NC State University Institutional Animal Care and Use Committee (IACUC) ID# 18-084-B (approval date: 25. May 2018).

### Complete blood counts, and isolation of serum and peripheral blood mononuclear cells

Blood was collected in three ways: (1) into SST tubes for serum collection, (2) into EDTA tubes for performing a complete blood count (CBC), and (3) into heparin tubes for the isolation of peripheral blood mononuclear cells (PBMC) (all tubes from BD Biosciences, San Jose, CA, USA). Serum SST tubes were incubated for 2 h, spun at 2,000 g for 20 min at 23°C; then, serum was collected and stored in aliquots at −80°C. The complete blood counts (CBCs) were determined using a Hemavet 950 system (Drew Scientific Group, Miami Lakes, FL, USA). The PBMC isolation was performed by density centrifugation using Ficoll-Paque-Premium (GE Healthcare, Uppsala, Sweden) in SepMate tubes (Stemcell Technologies, Vancouver, Canada). After isolation, PBMCs were frozen in 10% DMSO, 40% FBS, and 50% RPMI-1640 and either used fresh for *in vitro* restimulation assays or stored in liquid nitrogen for further downstream analysis.

### Esophageal endoscopy

The upper endoscope (GIF140; Olympus, Center Valley, PA) was advanced from the mouth to the proximal stomach using standard technique. The mucosa was examined carefully after any debris was washed away, and findings were recorded from three esophageal levels (distal, mid, and proximal). While edema was only present in one animal and rings and strictures were completely absent, gross pathological findings included white exudates, and furrows: these frequent findings were graded semi-quantitatively as mild (+), moderate (++), or strong (+++). Of note, the endoscopists (ESD, AB) were blinded as to which group (CON, Sens, Chall, S + C) the swine were in during the endoscopies. After the examination, four mucosal biopsies were taken from each of the three esophageal levels using large capacity forceps (RJ4; Boston Scientific, Maple Grove, MN). From each level, two biopsies were placed in formalin, one was placed in RNA later, and one was flash frozen for the following analyses.

### H + E and EPX stainings of esophageal tissue

Esophageal biopsies and tissue sections were fixed in 4% formalin (Thermo Fisher Scientific, Waltham, MA) for 24 h; then, the samples were transferred to 70% ethanol and stained with hematoxylin and eosin (H&E). Microscopic images were taken on a BX41 light microscope (Olympus, JPN) equipped with a high-resolution 14MP MU1400B digital camera imaging system and the AmScope v4.8 image analysis software (AmScope, ToupTek Photonics, CN). Stained tissue samples were scored by a veterinary pathologist who was blinded to experimental groups. Based on the criteria to score human EoE, eosinophil infiltration was quantified per histologic section of esophagus with a high-power field (HPF) section size of 0.24 mm^2^, and the peak eosinophil count was recorded. Of note, a peak number of 15 eosinophils per HPF is used to diagnose human EoE (44).

Eosinophil peroxidase (EPX) immunohistochemistry (IHC) was performed, as previously described (22, 45–48) with minor modifications for porcine tissue. Slides were deparaffinized, rehydrated, and heat induced epitope retrieval was performed using citrate buffer pH 6.0 (H-3300; Vector). Slides were blocked with Dual Endogenous Enzyme Block (S2003; Dako) for 10 min, followed by 2.5% normal horse serum (S-2012; Vector) for 30 min. Mouse monoclonal anti-EPX antibody (10 μg/ml, Lee, Jacobsen, MM25.82.2.1) was applied and slides were incubated for 1.5 h at room temperature. Slides were rinsed and anti-mouse HRP secondary antibody (MP-7402; Vector) was applied for 30 min. Visualization of EPX staining was performed using DAB chromogen (SK-4100; Vector). Slides were counterstained with Methyl Green (S1962; Dako). EPX-stained slides were then digitized (Aperio AT Turbo) and the number of EPX positive cells in an area equivalent to a HPF (0.24 mm^2^) were manually counted using Aperio ImageScope software (version 11.2.0.780). The HPF with the highest density of EPX positive cells was selected manually and reported as peak EPX-positive cells/0.24 mm^2^. EPX quantification was performed in a blinded manner.

### RNA isolation and quality control

Distal, middle, and proximal sections from porcine esophagi were extracted during necropsy. Tissue sections were preserved in Tri-Reagent (Invitrogen, Waltham, MA, USA) and stored at −80°C until further processing. Total RNA was isolated using the Direct-zol RNA Miniprep Kit (Zymo Research, Irvine, CA) following the manufacturer's instructions. RNA concentration and purity was measured using a Nanodrop 2000C spectrophotometer (Thermo Fisher Scientific). RNA size and quality were determined using a BioAnalyzer at the NCSU-Genomic Sciences Laboratory. All samples had a RIN >5 with an average RIN value of 6.9.

### RNAseq analysis on esophageal tissue and evaluation of immune cell activities with the CIBERSORTx database

RNA samples were submitted to the CGIBD Core of UNC Chapel Hill for library preparation and sequencing. cDNA library construction was carried out using the NEBNext Ultra II Directional RNA Library Prep Kit for Illumina (New England Biolabs, Ipswich, MA) following the manufacturer's instructions. Sequencing of the cDNA Libraries was performed with the NextSeq 500 (Illumina Inc., San Diego, CA). On average, approximately 60 million 75 bp paired-end reads per sample were generated. The raw data in FASTQ files were submitted to the Sequence Read Archive database (SRA) of the National Center for Biotechnology Information (NCBI) under the accession number PRJNA868364.

Nucleotides below Q25 or reads containing more than two ambiguous nucleotides were removed before sequence alignments performed by the CLC Genomics Workbench version 20.01 (QIAGEN Bioinformatics, Redwood City, CA). Reads were first mapped to a custom, manually-curated non-redundant (NR) 12,955 gene library for gene expression calculation. The sequences of the NR genes are in the Porcine Translational Research Database (PTR, https://www.ars.usda.gov/northeast-area/beltsville-md-bhnrc/beltsville-human-nutrition-research-center/diet-genomics-and-immunology-laboratory/docs/dgil-porcine-translational-research-database/). The remaining unmapped reads were subsequently mapped against the Ensembl pig genome build 11.1 (WG) in search of expressed genes that were not covered by the PTR database.

Transcriptomes built from the mapping results were subjected to differential expression analysis. The statistical analyses were carried out with the exact tests from the Bioconductor package “edgeR” (version 3.30.0; run on RStudio, version 4.0.3, Boston, MA) (49). Genes were considered differentially expressed with the thresholds of a false discovery rate (FDR) ≤0.05 and an absolute fold change ≥1.5.

For the evaluation of immune cells activities with the CIBERSORTx database (https://cibersortx.stanford.edu/), the proportions of 22 immune cells in each treatment groups were imputed from the transcriptome profiles with the CIBERSORTx tool (50). To evaluate the treatment effects on these results, ANOVA analyses were performed with the JMP Genomics (version 9, Cary, NC).

### Esophageal eotaxin RNA expression analysis *via* qPCR

RNA samples were reverse transcribed using the High-Capacity cDNA reverse transcription kit according to the manufacturer's instructions (Thermo Fisher Scientific). cDNA was synthetized from 100 ng RNA using a SimpliAmp thermal cycler (Thermo Fisher Scientific). The qPCR reaction was performed *via* the Quanta bio Perfecta SYBR Green FastMix (Quanta BioSciences, Beverly Hills, CA) using a qTOWER3G Real-Time qPCR thermocycler (Analytik Jena, Jena, GER). The following primers were used: Eotaxin-1: FWD 5′ GAT CCC CAC TCA GCG ACT AC 3', REV 5′ GAT CAC AGC ATT CTG GGG ACA 3′; Eotaxin-2: FWD 5′ GTG ATC TTC ACC ACC AGG AA 3', REV 5′ GAT CCT AGT GGA GGC TTT CTT C 3′; Eotaxin-3: FWD 5′ CAA GTT CTG CTG CTT CCA ATA C 3', REV 5′ GGT GGT GAA TAT CAC AGC CT 3′. The cycle conditions were as follows: 95°C for 3 min, 95°C for 10 s, 60°C for 30 s. The RPL19 gene was used as housekeeping gene.

### The OVA-specific CD4 T-cell response: *In vitro* restimulation and flow cytometry

For the analysis of the CD4 T-cell response, PBMCs were stained with CellTrace™ Violet proliferation dye (ThermoFisher Scientific, Waltham, MA, USA). Then, stained PBMC were cultured in 96-well plates at 2 × 10^5^ cells/well in RPMI-1640 (Corning, Corning, NY, USA) supplemented with 10% FBS (VWR, Radnor, PA, USA) and 1x antibiotic-antimycotic (Corning) in the absence or presence of 50 μg/mL OVA. After 4 days of culture, octuplicates were pooled and stained for flow cytometry analysis according to Table 1. Data were acquired on a Beckman Coulter CytoFlex using the CytExpert software (Beckman Coulter, Brea, CA, USA). Data analysis was performed using FlowJo v10.5.3 (FLOWJO LLC, Ashland, OR, USA).

**Table 1:** Flow cytometry antibody staining panel.

Antigen	Clone	Isotype	Fluorochrome	Labeling strategy	Primary Ab source	2nd Ab source
CD3	PPT3	IgG1	FITC	Directly conjugated	Southern Biotech	-
CD4	74-12-4	IgG2b	Brilliant Violet 480	Secondary antibody	BEI Resources	Jackson Immunoresearch
CD8α-biot	76-2-11	IgG2a	SA-BV605	Secondary antibody	BEI Resources	Jackson Immunoresearch
CCR7	3D12	rIgG2a	Brilliant Blue 700	Directly conjugated	BD Biosciences	-
GATA-3	TWAJ	IgG2b	R-PE	Directly conjugated	eBioscience	-
CellTrace	-	-	Violet (BV421 channel)	-	ThermoFisher	-
Live/Dead	-	-	Near Infra-red	-	Invitrogen	-

### Quantification of the OVA-specific serum IgG and IgE levels

Serum IgG and IgE levels were determined by ELISA. Nunc MaxiSorp™ flat-bottom 96 well plates (ThermoFisher Scientific, Waltham, MA, USA) were coated with 10 µg/ml ovalbumin and incubated at 4°C overnight. The plate was then washed twice with PBS + 0.05% Tween 20 (PBS-Tween, ThermoFisher Scientific, Waltham, MA, USA). The plates were blocked with 1% bovine serum albumin (Sigma Aldrich, St. Louis, MO, USA) in PBS for 1 h, followed by one wash with PBS-Tween. Serum dilutions were added in duplicates to the wells and incubated at 4°C overnight, followed by four washes with PBS-Tween. For the IgG ELISA, secondary rabbit anti-pig IgG conjugated with horse radish peroxidase (HRP, Invitrogen) was then added at 1:50,000 dilution for 2 h, followed by four washes with PBS-Tween. For the IgE ELISA, mouse anti-porcine IgE (Cloud-Clone, Katy, TX, USA) was then added at 1:10,000 dilution and incubated at 4°C overnight, followed by four washes with PBS-Tween. Rabbit anti-mouse IgG secondary antibody conjugated with HRP (ThermoFisher Scientific, Waltham, MA, USA) was then added at 1:50,000 dilution for 2 h, followed by four washes with PBS-Tween. The 3,3′,5,5′-Tetramethylbenzidine substrate solution (TMB, ThermoFisher Scientific, Waltham, MA, USA) was added for 30 min at room temperature in the dark. 1N HCl (ThermoFisher Scientific, Waltham, MA, USA) was added as a stop solution and data was recorded using a Synergy 2 Multi-Detection Micro Plate Reader (Biotek Instruments, Winooski, VT, USA) at 450 nm. Data are given as Optical Density (OD) values.

### Statistical analysis

Statistical analysis was performed using GraphPad Prism 9.4.0 (GraphPad Software, San Diego, CA). Depending on the dataset, statistical significance was analyzed using (i) a mixed-effects model with Geisser-Greenhouse correction and a Šídák's multiple comparisons test (CD4 and IgE response), (ii) a non-parametric Mann-Whitney test (esophageal eotaxin expression), or (iii) an unpaired *t* test (Esophageal eosinophilia and EPX^+^ cells). For the RNAseq analysis, the statistical analyses were carried out with the exact tests from the Bioconductor package “edgeR” (version 3.30.0; run on RStudio, version 4.0.3, Boston, MA). Genes were considered differentially expressed with the thresholds of a false discovery rate (FDR) ≤0.05 and an absolute fold change ≥1.5. The performed statistical tests are also indicated in the respective figure legends.

## Results

**Figure 2:**
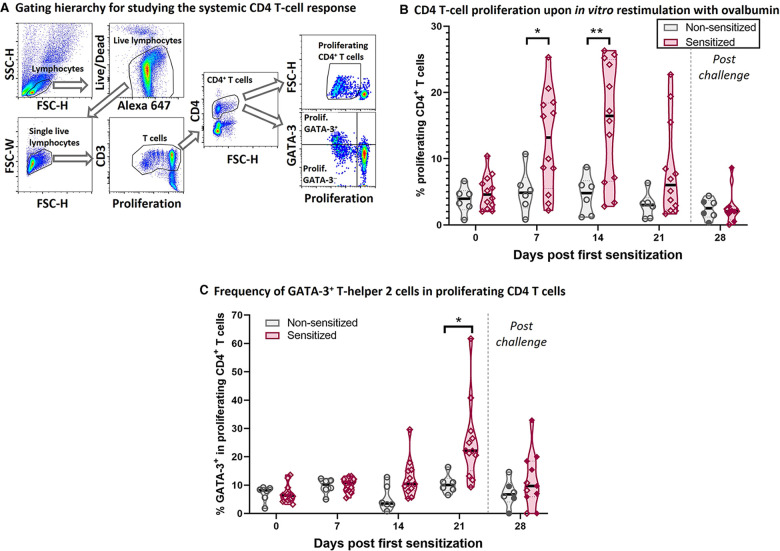
HEWP sensitized swine develop a systemic T-helper 2 response. PBMC from sensitized and non-sensitized swine were isolated, stained with a violet proliferation dye, and cultured *in vitro* in the presence of 50 µg/ml ovalbumin. At the end of the culture, PBMC were stained for multi-color flow cytometry analysis according to Table 1. (**A**) Shows the gating hierarchy to assess the proliferative CD4 T-cell response to ovalbumin. Lymphocytes were selected using a FSC-H/SSC-H gate. A live/dead discrimination dye was included to exclude dead lymphocytes. Doublets were excluded using a FSC-width (FSC-H)/FSC-area (FSC-W) gate on singlets. Single live lymphocytes were then used to identify CD3^+^ T cells (proliferation dye/CD3), and then CD4^+^ T cells. The proliferation of these CD4 T cells was then analyzed using a violet proliferation dye (Proliferation/FSC-H). In addition, the frequency of GATA-3^+^ cells within the proliferating CD4 T cells was analyzed using a Proliferation/GATA-3 quadrant. (**B**) Shows the frequency of proliferating CD4 T cells at different days after their first sensitization (dps). (**C**) Shows frequency of GATA-3^+^ cells within the proliferating CD4 T cells. Of note, the 21 and 28 dps data were obtained using frozen samples and the 28 dps represents the time point after the 7 days of oral challenge. Reactivity of frozen PBMC was confirmed by ConA stimulation that induced highly similar proliferative responses to freshly isolated PBMC (data not shown). Grey violin plots represent non-sensitized animals; purple violin plots show sensitized animals. Individual data points are shown in circles (non-sensitized), or diamonds (sensitized). The filled symbols at 28 dps represent the animals received oral challenge. Data were statistically analyzed using a mixed-effects model with Geisser-Greenhouse correction and a Šídák's multiple comparisons test. **p < 0.01, *p < 0.05.

**Figure 3:**
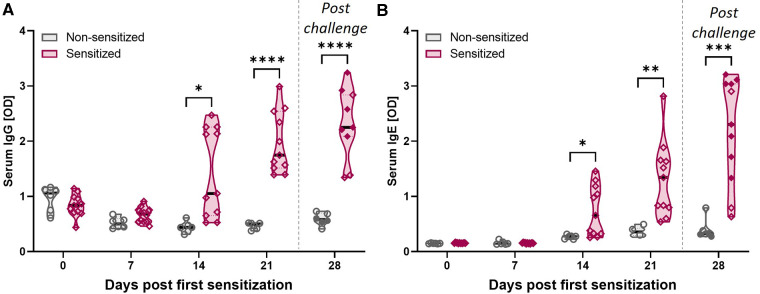
HEWP sensitization increases the OVA-specific serum IgG and IgE levels. OVA-specific IgG (**A**) and IgE (**B**) levels in serum at different time points (0–28 days post sensitization) were analyzed by ELISA. Data are shown as optical density (OD) values. Grey violin plots represent non-sensitized animals; purple violin plots show sensitized animals. Individual data points are shown in circles (non-sensitized), or diamonds (sensitized). The filled symbols at 28 dps represent animals that received oral challenge. Data were statistically analyzed using a mixed-effects model with Geisser-Greenhouse correction and a Šídák's multiple comparisons test. ****p < 0.0001, ***p < 0.005, **p < 0.01, *p < 0.05.

**Figure 4:**
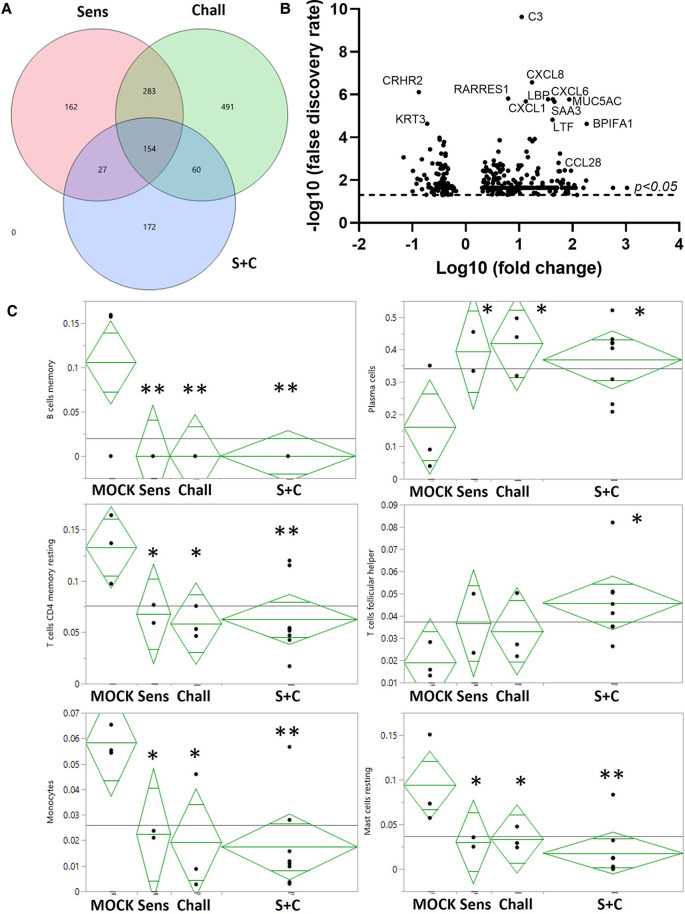
RNA expression changes induced by intraperitoneal sensitization and oral challenge. Esophageal tissue samples were collected during necropsy to isolate total RNA. Global gene expression was analyzed *via* RNAseq. (**A**) Summary of the EDGE R analysis: the overlaying circles show the number of differentially expressed (DE) genes between non-sensitized/non-challenged control animals (CON), and sensitized (Sens, red), challenged (Chall, green), and/or sensitized and challenged (S + C) swine. (**B**) The volcano plot shows the global DE genes (*p* < 0.05) between CON and S + C swine. Each circle represents once gene. The x-axis delineates the fold changes (log10) and the *y*-axis the −log10 of the false discovery rate (FDR). The top12 differentially expressed genes (by FDR) plus the eosinophil-attracting chemokine CCL28 are identified. (**C**) The proportions of 6/22 immune cells in each treatment group were imputed from the transcriptome profiles with the CIBERSORTx tool (50). To evaluate the treatment effects on these results, ANOVA analyses were performed with JMP Genomics (version 9, Cary, NC). **p < 0.01, *p < 0.05.

**Figure 5:**
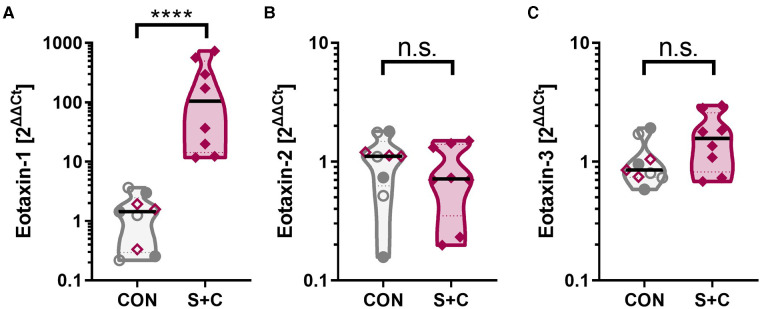
Intraperitoneal sensitization and oral challenge increases the esophageal eotaxin-1 expression. Esophageal tissue samples were collected during necropsy; RNA was extracted, and eotaxin-1 (=CCL11) mRNA expression analyzed by RT-qPCR. GAPDH was used as housekeeping gene and data are expressed as 2^ΔΔCt^. Control animals consist of control swine (CON, open grey circles), challenged swine (Chall, filled grey circles), or sensitized swine (Sens, open purple diamonds). The S + C swine are represented by the filled purple diamonds. Data were statistically analyzed using a non-parametric Mann-Whitney test. ****p < 0.0001.

**Figure 6:**
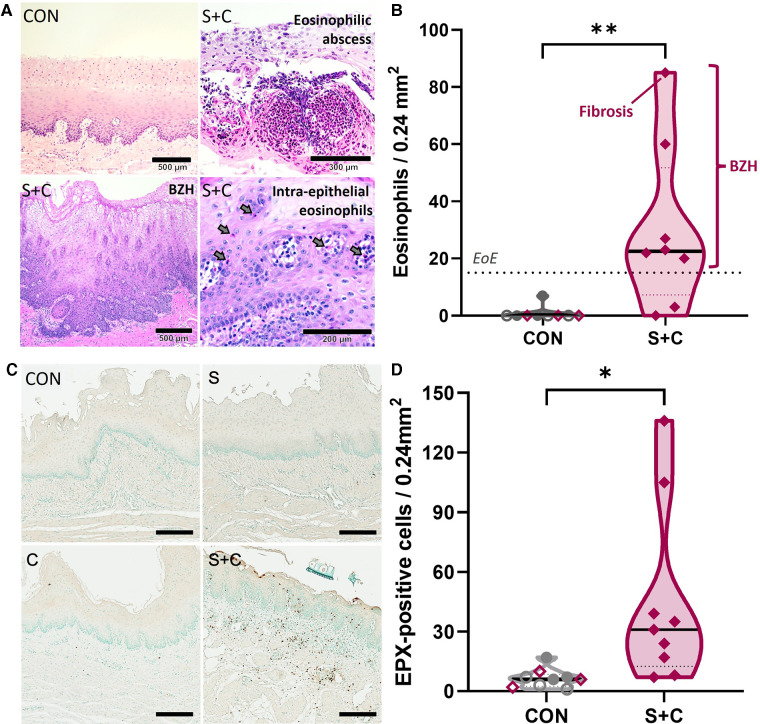
HEWP sensitized and orally challenged swine develop esophageal eosinophilia, show increased eosinophil activity, and histopathological changes associated with eosinophilic esophagitis. Esophageal histology samples taken during necropsy were stained with H + E (**A,B**) or for eosinophil peroxidase (EPX, **C,D**). (**A**) Original H + E images of one control (CON) and three selected sensitized and orally (S + C) challenged swine. The chosen images show representative findings of the trial—intraepithelial eosinophils (including an eosinophilic abscess), BZH, fibrosis. (**B**) Quantitative analysis of the eosinophil infiltration showing the peak eosinophils/0.24 mm^2^; this corresponds to the quantification used to diagnose human EoE patients. The dotted line at 15 eosinophils/0.24 mm^2^ indicates the corresponding threshold for this diagnosis. The sensitized and orally challenged (Sens + chall) swine shown in (**A**) are identified by swine number (#); swine with fibrosis and BZH are additionally highlighted. Data were statistically analyzed using an unpaired *t* test. **p < 0.01. (**C**) EPX IHC staining of representative CON, sens, chall, and S + C swine. (**D**) Quantitative analysis of the peak number of EPX positive cells per tissue area (0.24 mm^2^). Data were statistically analyzed using an unpaired *t* test. **p < 0.01.

**Figure 7:**
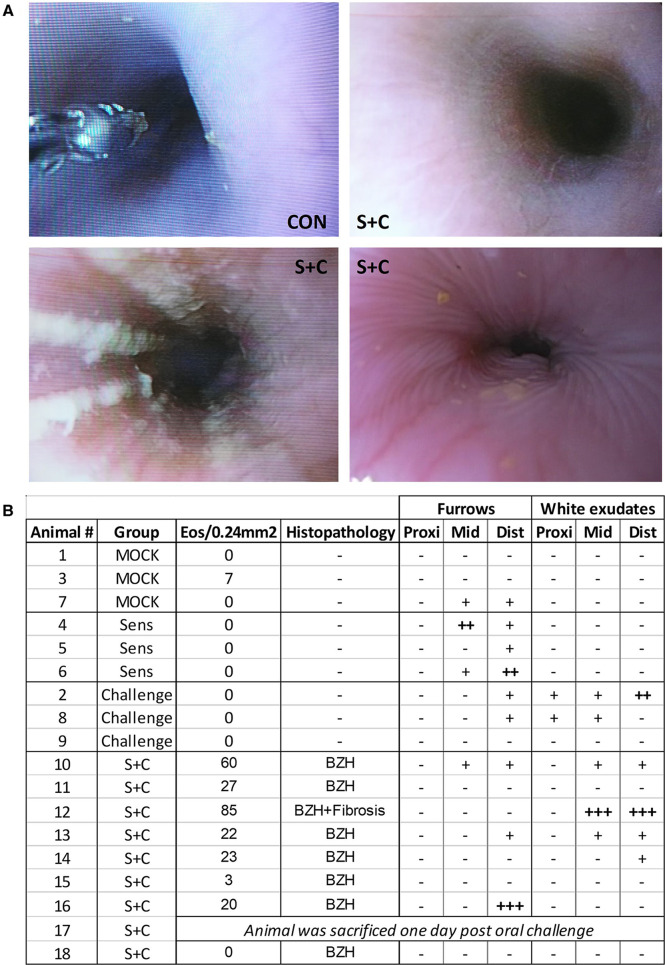
HEWP sensitized and orally challenged swine develop grosspathological changes associated with eosinophilic esophagitis. Swine underwent endoscopy with an Olympus GIF140 upper endoscope, a model that has also been used for humans. (**A**) Original endoscopy images from one control swine (CON) and three sensitized and challenged swine (S + C swine). The chosen images show representative findings of the endoscopy with linear furrows and white exudates. (**B**) Tabular presentation of both the histologic and endoscopic findings that are mainly associated with human EoE linear furrows and white exudates in the proximal (prox), mid, and distal (dist) parts of the esophagus.

In a previous study, we provided evidence that intraperitoneal administration with HEWP and cholera toxin followed by oral challenge of HEWP-induced esophageal eosinophilia (33). The two goals of this study were to demonstrate that sensitization and oral challenge not only induces the immune mechanisms underlying human EoE (Figures 2–5) but also leads to its esophageal histologic and in some animals also gross pathologic changes (Figures 6, 7).

### The systemic OVA-specific T-helper 2 response

To better understand the underlying immune mechanisms of the Th2-driven food allergy EoE, we first analyzed the systemic anti-OVA CD4 T-cell response (Figure 2). Figure 2A shows the flow cytometry gating hierarchy used to study the proliferation and GATA-3 expression of CD4 T cells after 4-day *in vitro* restimulation with OVA: After gating on porcine lymphocytes and excluding both dead cells and doublets, the gated single live lymphocytes were first gated on T cells (Proliferation/CD3) and then on CD4 T cells (FSC-H/CD4). These CD4 T cells were then analyzed on their proliferative response to OVA (Proliferation/FSC-H); the proliferating CD4 T cells were then additionally analyzed on their expression of the Th2 marker GATA-3 (Proliferation/GATA-3) (Figure 2A). Compared to non-sensitized swine, CD4 T cells from sensitized swine proliferated significantly stronger at 7- and 14-days post (first) sensitization (dps, Figure 2B). Proliferating CD4 T cells in sensitized animals showed an increased expression of the Th2-associated transcription factor GATA-3 at 14 dps (non-significantly) and 21 dps (Figure 2C). The combination of the strong systemic CD4 T-cell response and the GATA-3 upregulation demonstrates that intraperitoneal sensitization with HEWP and cholera toxin induces a systemic Th2 response—the underlying T-cell response of human EoE.

### The systemic OVA-specific IgG and IgE response

CD4 T cells are important activators of the humoral immune response including the production of both IgG and IgE. In addition, while EoE is mainly Th2-driven, EoE has also been reported to be a “mixed IgE- and non-IgE-mediated allergic response” (51). Hence, OVA-specific IgG and IgE levels were quantified in serum (Figure 3). Starting at 14 dps, both anti-OVA serum IgG (Figure 3A) and IgE (Figure 3B) levels were significantly elevated in HEWP-sensitized animals. In addition, post challenge (=28 dps), two of the three swine within the sensitized group that did not receive oral challenge (open diamonds) had the lowest anti-OVA serum IgG and IgE levels. Therefore, HEWP-sensitization induced a systemic anti-OVA IgG and IgE response that seems to be further elevated by HEWP oral challenge.

### The esophageal immune profile

To provide a broad understanding of the immunological changes induced by the HEWP sensitization and challenge, the esophageal immune profile was analyzed by RNAseq (Figure 4). All treatments, (sensitization, challenge, and sensitization + challenge) induced hundreds of RNA expression changes—some shared, some unique (Figure 4A). Most importantly for our model, compared to CON swine, the sensitized and challenged swine showed a total of 413 differentially expressed (DE) genes (Figure 4A, Supplementary Table S1). Within these, 113 genes (27.8%) have been previously associated with food allergy; within the top 10 DE genes, 8 genes have been previously linked to food allergy—C3, CXCL8, RARRES1, CXCL6, MUC5AC, CXCL1, and SAA3 (Figure 4B). Therefore, the most affected immune parameters are related to (i) the complement system [C3 (52)], (ii) chemokine signaling (CXCL-1, -6, and -8), (iii) gel-forming mucin production [MUC5AC (53)], and (iv) a protein with various and controversially discussed functions—SAA3 (54, 55).

The obtained RNAseq data were additionally analyzed by the CIBERSORTx software (Stanford University, Stanford, CA, USA). CIBERSORTx estimates the abundance of different cell types such as immune cell subsets. Compared to esophageal tissue from CON animals, CIBERSORTx estimated changes in B/plasma cells, T cells, and myeloid cells: (i) while resting memory B cell numbers were reduced, the plasma cells were more abundant; (ii) resting memory CD4 T cells were also less prevalent, follicular-helper CD4 T cell (Tfh) numbers were increased; (iii) within the myeloid cells, both monocytes and resting mast cells were less prevalent (Figure 4C). In summary, the identified differences show an estimated decrease in resting immune cells and monocytes but an estimated increase in active effector cells from the adaptive immune system.

### Eotaxin expression in the esophagus

In addition to the broad RNAseq analysis, a specific RNA expression analysis has been performed *via* qPCR for the chemokine family that attracts eosinophils and is crucially associated with EoE—eotaxins [eotaxin 1, 2, and 3 (56)]. While eotaxin-2 (=CCL24) and eotaxin-3 (=CCL26) levels were unaltered between control (CON, Sens, and Chall swine) and the S + C swine, esophageal eotaxin-1 (=CCL1) levels were significantly elevated in S + C swine (Figure 5). So, while human EoE patients mainly show elevated eotaxin-3 levels (56), in swine, HEWP-sensitization and oral challenge induced an esophageal expression of another eosinophil-attracting chemokine—eotaxin-1.

### Esophageal eosinophilia, eosinophil peroxidase production, and histopathology

Increased eotaxin-1 levels indicate that eosinophils are being recruited into the esophagi of sensitized swine. To quantify eosinophils and to demonstrate eosinophilia and other EoE-associated histopathological changes within the esophagus, esophageal tissue samples collected during necropsy were stained with H + E (Figures 6A,B). Representative H + E images from a CON and three S + C swine demonstrate histopathological changes associated with EoE such as substantial eosinophil infiltration, an eosinophilic abscess, basal zone hyperplasia (BZH), and fibrosis (Figure 6A). While there were no changes in the eosinophil numbers in peripheral blood (Supplementary Figure S1), quantification of esophageal eosinophil infiltration (Figure 6B) showed that in contrast to control animals (CON, Sens, and Chall), 6/8 S + C swine showed esophageal eosinophil infiltrations of >15 eosinophils/0.24 mm^2^—the threshold for human EoE diagnosis (51). In addition, the esophageal eosinophilia was associated with concomitant basal zone hyperplasia and the swine with the highest eosinophil count even showed signs of subepithelial fibrosis.

To better demonstrate the extent of eosinophilia in the revealed histopathological changes, eosinophils were additionally investigated by staining for the eosinophil secondary granule protein eosinophil peroxidase (EPX, Figure 6C,D). Representative EPX staining from CON, Sens, Chall, and S + C esophageal tissue sections are shown. Extensive EPX staining in the S + C animals clearly outlines the eosinophilic infiltration (Figure 6C). The quantification and statistical analysis of EPX^+^ cells show a significant increase in EPX-producing cells within the esophagi of S + C swine (Figure 6D). Thus, S + C swine showed most major histopathological hallmarks of human EoE—esophageal eosinophilia, basal zone hyperplasia, and in one instance fibrosis.

### Gross pathological changes associated with EoE: White exudates and linear furrows

In addition to histopathological changes, gross pathological changes were monitored *via* endoscopy at the end of the sensitization and oral challenge period (Figure 7). The representative images in Figure 7A show a healthy swine esophagus (CON) as well as the esophagi from three S + C swine. Compared to the healthy esophagus of the CON swine, some S + C swine developed white exudates and/or linear furrows. Of note, the swine (#12) with the highest eosinophil numbers and signs of fibrosis (Figures 6B, 7B: Animal 12) also presented the strongest gross pathological changes—substantial white exudates and linear furrows (Figure 7A, bottom left picture). However, not every swine showed clear gross pathological signs of EoE. After the triple EoE sensitization and 1-week of oral challenge, our outbred swine model provided various degrees of gross pathological changes (Figure 7B): changes ranged from no pathology (3/8 swine) over mild changes (3/8 swine) to strong linear furrows (1/8 swine) and white exudates (1/8 swine). In some ways, this mimics the variability of endoscopic signs seen in human EoE (57).

In summary, the triple weekly intraperitoneal sensitization followed by 1 week of daily oral challenge in the S + C group induced immune mechanisms and pathological changes as observed in human EoE patients: Sensitization led to a systemic Th2 response as well as elevated anti-OVA serum IgE levels; the combined sensitization and oral challenge protocol was required to induce the histo- and gross-pathological changes used to histopathologically diagnose human EoE—eosinophilia, basal zone hyperplasia, and esophageal fibrosis. While mild gross pathological changes also could be observed in CON, Sens, and Chall swine, the strongest signs of EoE were found in the S + C groups—linear furrows, and/or white exudates.

## Discussion

Swine have been used as biomedical animal models in a variety of research areas and diseases such as pre- and post-natal development and aging, allergy, obesity and nutrition, cancer, infectious diseases, vaccine development, and allergy including food allergy [reviewed in (37, 38)]. Research in swine on egg food allergy has been driven by the Wilkie group (42, 58–61). In addition, Helm et al. (62) and more recently Mondoulet et al. (63) have used the swine model to study peanut allergy. These studies demonstrate that swine can be used as a biomedical animal model for food allergy research. However, so far only one study showed that swine can develop esophageal eosinophilia (33). This was the first sign that swine could be used as a biomedical animal model for EoE. Based on the high relevance of the swine model for biomedical research and the previous successes in modeling allergic diseases, and the finding that swine can develop esophageal eosinophilia, this study sought to fully develop swine as a novel biomedical animal model for translational research on EoE. To this end, we not only demonstrated the development of esophageal eosinophilia and histopathological changes but also determined the systemic and local underlying immune mechanisms as well as the gross pathological outcomes.

Our data demonstrate that intraperitoneal sensitization was the main driver of the systemic Th2 response: at 7 and 14 dps, CD4 T cells demonstrated a strong proliferative response to the food allergen OVA; shortly after (at 21 dps), these proliferating CD4 T cells expressed elevated levels of the Th2 marker GATA-3 (Figure 2). These data underscore that the systemic Th2 response is a crucial event in the initiation of EoE (64). However, the one week of oral challenge was not able to further increase the systemic Th2 response. Nevertheless, while this proliferative Th2 response was less intense, it cannot be ruled out that the systemic Th2 response is involved in maintaining the allergic response. The Rothenberg laboratory demonstrated in 2007 that compared to nonatopic control children, EoE patients with active disease had increased levels of systemic IL-5 producing CD4 T cells (65). Based on the differentiation process of CD4 T cells (66), allergen-specific CD4 T cells are activated and start to proliferate before they differentiate into effector cells. These effector CD4 T cells shift their activity from proliferation to an effector function—like the production of cytokines such as IL-4, -5, and -13. Hence, while a proliferative Th2 response might be essential in the initiation of EoE, the systemic cytokine producing Th2 cells might be the immune mechanisms responsible for maintaining and driving long-term EoE. With the constant increase in the immunological toolbox for swine, novel tools like an anti-swine IL-5 antibody might soon become available to enable the quantification of IL-5 producing Th2 cells in swine. In combination with a long-term oral challenge phase, this tool would facilitate monitoring CD4 T-cell activity and differentiation during the onset and active phases of EoE in swine.

An indication that the Th2 response might continue past the sensitization phase is the ongoing and elevated anti-OVA IgE levels in the serum of S + C swine (Figure 3). CD4 T cells are crucially involved in the activation of B cells, their differentiation into memory B cells and antibody producing plasma cells, as well as in isotype switching. Besides IL-5, Th2 cells also produce IL-4; this cytokine regulates isotype switching in B cells towards the isotypes IgG1 and IgE (67). Serum anti-OVA IgG and IgE levels were increased in S + C swine during both the sensitization and oral challenge phase. While we showed that sensitization and challenge increased the OVA-specific serum IgG levels, we could not identify the IgG subtype of these anti-OVA antibodies. Hence, the establishment of an anti-OVA IgG1-specific ELISA is necessary to serve as an indirect measure of the systemic Th2 response. However, we could also show a steady increase in IgE levels beyond the sensitization phase. This observation indicates that Th2 cells might still be active during the oral challenge phase. However, since CD4 proliferation precedes antibody production, more long-term studies are needed to confirm the indication that IL-4 producing Th2 cells drive the IgE production not only during the onset but also the long-term active phases of EoE.

In addition to being an indicator for a Th2 response, IgE, while not being the mediator, could still play a role in EoE. This role of IgE in EoE has been well reviewed by Simon et al. in 2016 (68). EoE patients had elevated total and food- and aeroallergen-specific IgE levels (69). In addition, in 91% of adult EoE patients, IgEs to food- and inhalant allergens have been detected (70). Additionally, immunoglobulin class switching to IgE has been described in the esophagi of pediatric EoE patients; the local production of IgE strongly indicated a role of IgE in EoE (71). Based on the combination of those observations, the initial assumption was that EoE is an IgE-mediated food allergy (68). However, other studies challenged this assumption and currently IgE is not considered to drive EoE. Allergen-specific IgE levels and skin prick testing did not conclusively identify the underlying food allergens (70, 72–74). In turn, IgE-sensitization-based food elimination diets could not significantly reduce the number of EoE patients with average predictive values for food allergens of 47% (74). Furthermore, while an anti-IgE antibody therapy significantly reduced IgE levels in esophageal tissue, it was not effective as compared to placebo in a randomized clinical trial (75, 76). Based on these controversial outcomes, we can conclude that while IgE does not seem to be the (sole) driver of EoE, IgE seems at least to be associated with EoE. In the current study, the swine in the S + C group, not only showed the highest esophageal eosinophil numbers, but with one exception, these swine also had by far the highest serum anti-OVA IgE levels (Figures 3, 28 dps, filled red diamonds). So, while we cannot answer the causative nature of IgE in EoE, our data from this model further support at least a correlation between EoE and systemic anti-allergen IgE levels.

In addition to the systemic CD4 T-cell and antibody responses, the local immune response in the esophagus was analyzed by RNAseq (Figure 4). While RNAseq data on its own is not adequate to conclude on mechanisms of food allergy and EoE, it can be used to generate hypotheses that inform future studies on food allergy/EoE in swine. Within the 413 DE genes between the S + C and control group, the complement factor C3, and the chemokines CXCL-1, -6, and -8 belonged to the most upregulated DE genes. These DE genes encompass immune genes involved in the early phases of an immune response—induction of inflammation and cell recruitment. RNAseq studies using human esophageal biopsy samples from healthy donors and EoE patients identified *ALOX15*, *CCL26*, *CLC*, and *CPA3* as well as the long non-coding RNA BRAF-activated non-coding RNA (BANCR) as the most upregulated genes and *ALOX15*, *CCL26*, *CLC*, and *CPA3* as strongly downregulated genes (77). However, except for CCL26, the most DE genes do not belong to the chemokine or complement families. This discrepancy can be best explained by the different timelines: while in this study, swine were challenged for only one week, human EoE patients will seek diagnosis and/or treatment at a much later timepoint. Therefore, the DE expressed genes in this study can inform about the early stages of EoE; in contrast, human RNAseq data best reflect gene expression changes during established EoE.

In the following, we will therefore discuss the most significantly DE genes identified in this study with their known roles in (food) allergy and/or EoE. C3 is a central component of the complement system. It has previously been linked to asthma (78), associated with a Th2 response (79), and it is a strong positive regulator of inflammation (80). In addition, a study investigating the role of complement in food allergy-induced anaphylactic shock, showed that the C3 cleavage product, C3a, contributes to the peanut-induced shock by stimulating macrophages, basophils, and mast cells to produce inflammatory molecules like the platelet activating factor (PAF) and histamine (52). Inflammation can lead to vasodilation and increased vascular permeability facilitating the influx of immune molecules and cells. In addition, chemokines will direct specific immune cells to the site of infection. The chemokines CXCL-1, -6, and -8 that were upregulated in the S + C group, have also been linked to allergy (81–83). CXCL-6 and -8 bind to the chemokine receptors CXCR-1 and/or -2 that are mainly expressed on neutrophils; this binding can then lead to neutrophil-activating and angiogenic actions (84–86). Therefore, the complement C3 and the chemokines CXCL-1, -6, and -8 might be involved in the observed inflammatory process in the esophagi of S + C treated swine. Interestingly, eosinophils do not seem to express the CXCR-1 and -2 receptors for these most upregulated chemokines (87). Therefore, we included a specific qPCR expression analysis for eosinophil-attracting chemokines—eotaxins 1–3. This directed approach showed that in S + C treated swine, eotaxin-1 expression was significantly increased in S + C swine (Figure 5). While future studies still need to determine the effects of the different eotaxin usage between humans [eotaxin-3 (56)] and swine, this result supports and explains the observed increased presence and activity of eosinophils in the esophagi of S + C treated swine (Figure 6).

Potentially more downstream of the effects of C3 and chemokines are the other strongly DE genes that were upregulated in S + C swine and associated with allergy—the mucin MUC5AC, and the serum amyloid A 3 (SAA3). The production of the mucin MUC5AC is induced by retinoic acid (88) and IL-13 (89), and this secreted gel-forming mucin has previously been associated with a Th2 response (90) and allergic diseases such as asthma (91) and EoE (92). The serum amyloid A3 (SAA3) is the local and major acute phase SAA isoform in swine (93) and promotes atherosclerosis (94)—a mechanism that has been linked to IgE (95). Therefore, our RNAseq data further indicate that the S + C treatment induced not only inflammation and cell recruitment but also potentially more downstream effects on mucin production and an acute phase response. However, as mentioned above, the hypothesized implications of these RNAseq results need to be confirmed in future studies. Taken together, both the systemic Th2 and IgE responses as well as the immune mechanisms in the esophagi of S + C treated swine show strong similarities to the immune mechanisms observed in human allergy and EoE.

In addition to the systemic and local immune mechanisms, the histologic and gross pathologic changes in S + C treated swine were monitored *via* histology and endoscopy respectively. In contrast to the systemic immune responses that were mainly driven by sensitization, local signs of EoE were only observed in the S + C group (Figures 2–6). As in human EoE patients, 6/8 S + C-treated swine developed the major diagnostic marker for EoE—esophageal eosinophilia [(51), >15 peak eosinophils/0.24 mm^2^, Figure 6]. Interestingly, all of these swine also developed basal zone hyperplasia that has been previously associated with human EoE (92). In addition, the S + C treated swine with the highest eosinophil infiltration also demonstrated signs of fibrosis. By causing esophageal dysmotility and strictures, fibrosis is a major consequence of untreated eosinophilic inflammation and leads to clinical complications of EoE (96). While the majority of S + C treated swine demonstrated clear histological signs of EoE, the gross pathological changes were more diverse. Notably, linear furrows could be observed throughout the treatment groups and in one instance, mild linear furrows were even noticed in a CON animal. However, white exudates only occurred in orally challenged and S + C swine (Figure 7). A potential explanation for the difference between the more frequent and unified histological changes and the diverse endoscopic changes could be that 3 weeks of sensitization followed by 1 week of oral challenge is sufficient to induce histological changes, but the gross pathological changes associated with EoE can take longer to develop. Hence, to study the full pathology including gross pathological changes in the esophagus, more long-term studies are necessary. Such long-term studies could also include more natural ways of EoE sensitization such as detergent treatment of the esophagus (97). The combination of the natural sensitizations and the long-term challenge could make swine an excellent model for studying the immune mechanisms and pathological changes during both, the onset and the long-term disease. Importantly however, the S + C swine with the highest eosinophil numbers, basal zone hyperplasia, and fibrosis also displayed the most pronounced gross endoscopic signs of EoE—linear furrows and prominent white exudates (Figure 7A, bottom left image, and Figure 7B, swine 12). These pathological data indicate that S + C treated swine show robust histopathological changes and gross pathological changes associated with EoE in humans.

## Summary and conclusion

The combined analysis of the systemic and local immune response demonstrates that the S + C treatment induces immune mechanisms previously associated with allergies and EoE in humans. Systemically, the S + C treatment induces an OVA-specific Th2 response (Figure 2) as well as elevated levels of anti-OVA IgE (Figure 3). Locally, the immune response in the esophagi of S + C treated swine indicated an increased complement and chemokine response; and it showed a strong infiltration of eosinophils. In addition to the EoE-related immune mechanisms, S + C treated swine also develop the histopathologic and endoscopic changes associated with EoE—esophageal eosinophilia, basal zone hyperplasia, and in some instances, fibrosis, linear furrows and white exudates. Based on the general immunological (37) and physiological similarities (30, 98) between swine and humans, the successful usage of swine as a biomedical animal model for allergies, including food allergy (33, 38, 42, 58–63, 99–101), and the similarities between the immune mechanisms and pathology of S + C treated swine and human EoE patients, we conclude that swine represents a valuable animal model for biomedical research on EoE. The logical next steps would be on the one side to perform long-term challenge studies to determine the immunological and pathological changes over time, and on the other side to test the responsiveness of EoE-swine to human treatment—e.g., steroid treatments or allergen avoidance.

## Data Availability

The datasets presented in this study can be found in online repositories. The names of the repository/repositories and accession number(s) can be found in the article/Supplementary Material.
