# Supplementary material for: Immunologic and pathologic characterization of a novel swine biomedical research model for eosinophilic esophagitis
Source: Front Allergy. 2022 Nov 14;3:1029184. doi: 10.3389/falgy.2022.1029184 (PMC9701751; doi:10.3389/falgy.2022.1029184)
Supplement: Supplementary file 1 [file Datasheet1.pdf]

Gene	Original Gene Annotation	FDR	Fold change	Association *	Annotations
**C3**	C3	2.35E-10	11.2	A	Asthma, C3 deficiency (LOF) (31953710); Asthma, C3 GOF (31953710); Classical and Alternative Complement Activation Pathways; Obesity (20105310); Positive Acute Phase Reactant;(20402389); Positive Regulator of Inflammation (18802120); Th1-Associated (16517700); Th2-Associated (12496433)
**CXCL8**	CXCL8	2.67E-07	17.4	A	Angiogenesis; Positive Regulator of Apoptosis; Asthma; CXCR1 Ligand; CXCR2 Ligand; Inflammation; Kinocidin (17208195); Negative Regulator of Cell Adhesion; Neutrophil Chemoattractant
**CRHR2**	CRHR2	7.72E-07	-7.5		Corticotropin Releasing Hormone Receptor
**RARRES1**	RARRES1	1.54E-06	6.3	A	Retinoic Acid Induced (8601727)
**LBP**	LBP	1.66E-06	34.9	A	Positive Acute Phase Reactant; LPS-binding Protein; Pattern Recognition Receptor; Toll-like Receptor Signaling Pathway
**CXCL6**	CXCL6	1.66E-06	42.7	A	Adipocytokine (21252989); Asthma (15585884); CXC Chemokine; Cytokine; CXCR1 Ligand; CXCR2 Ligand
**MUC5AC**	MUC5AC	1.68E-06	86.6	A	Asthma (19614611); IL- Induced (27588910); IL-13 Induced (27588910); Retinoic Acid Induced (17646388); Secreted Gel Forming Mucin; TH2-Associated (19718656); STAT6 Induced (27588910)
**CXCL1**	CXCL1	2.10E-06	13.3	A	Asthma (15585884); CXC Chemokine; Cytokine; CXCR2 Ligand; Kinocidin (17208195)
**SAA3**	SAA3	2.17E-06	46.0	A	Positive Acute Phase Reactant (21439655)
**LTF**	LTF	1.51E-05	42.0	I	Antibacterial Response (8423097); Iron Metabolism
**KRT3**	KRT3	2.28E-05	-5.3		
**BPIFA1**	BPIFA1	2.34E-05	184.2	I	Antibacterial Responses (21787346)
**CILP**	CILP	1.04E-04	-3.1		
**SPP1**	SPP1	1.20E-04	15.7	I	Cytokine (15855273); Estrogen Induced; Positive Regulator of Type 1 IFN Synthesis (16604075); Positive (chronic) or Negative (acute) Regulator of Intestinal Inflammation (18627421); TH1-Associated (11037970); Retinoic Acid Regulated (1633816); Vitamin D Regulated (1633816)
**CFTR**	CFTR	1.20E-04	19.5	A	Asthma (11344348); Chloride Ion Transport; Inflammation (21712022); Cystic Fibrosis (31953710); Th2-Associated (11777980)
**LPIN1**	LPIN1	1.28E-04	-3.1		
**LCP1**	LCP1	1.37E-04	4.3	A	Atopic Dermatitis (27304220); Positive Regulator of Eosinophil Activation (21525390); Positive Regulator of T Cell Activation (17294403)
**ALDH1A3**	ALDH1A3	1.57E-04	17.6	A	Biosynthesis of Retinoic Acid; Vitamin A Metabolism
**KLK9**	KLK9	1.65E-04	-2.5		Protease (28559305)
**PI16**	PI16	2.34E-04	-2.6	I	CD Marker; Treg T Cells (h) (20554955)
**KLK1**	KLK1	3.67E-04	-4.7		
**TRPV6**	TRPV6	4.67E-04	13.7	A	Chloride Ion Transport; Epithelial Cell Calcium Transporter (26109800); Vitamin D Induced (11545681)
**MPEG1**	MPEG1	4.67E-04	4.1	I	Antibacterial Responses (23257510); Type 1 IFN Induced (23257510); Type 2 IFN Induced (23257510)
**DUSP14**	DUSP14	5.81E-04	-2.9		Negative Regulator of Inflammation (29077210); Susceptibility to Tuberculosis (26938665)
**FBXO32**	FBXO32	5.81E-04	-2.4		
**IGKC**	ENSSSCG00000036224	5.81E-04	58.0	A	Immunoglobulin kappa constant chain
**PLCXD1**	PLCXD1	7.23E-04	-2.4		Phospholipase C
**KRT7**	KRT7	8.55E-04	14.6	A	Keratin; Goblet Cells (10892854)
**LOC106510150**	ENSSSCG00000049179	8.70E-04	-14.6		Long Non-coding RNA (LncRNA)
**C1QL4**	C1QL4	1.06E-03	-6.6		BAI3 Ligand (21262840); Negative Regulator of Adipogenesis (23449976)
**GIMAP5**	GIMAP5	1.23E-03	3.3	I	IAP and IAP-Related (15474311); T Cell Development (16509771)
**SLA-DQA1**	SLA-DQA1	1.48E-03	2.3	A	Antigen Presentation; Major Histocompatibility Complex (SLA) Class II; Positive Regulator of TCR Signaling
**CCL28**	CCL28	1.57E-03	54.9	A	Asthma (15681819); CCR3 Ligand (10975800); CCR10 Ligand (10975800); CCL Chemokine; Cytokine; Inflammation (15246961)
**SLC25A45**	SLC25A45	1.64E-03	-4.2		
**WNK4**	WNK4	1.73E-03	-3.3		
**CD83**	ENSSSCG00000021576	1.73E-03	4.4	A	B Cell Activation Marker (8422464); CD Marker; Cell Surface Protein; Dendritic Cell Maturation Marker (12403928)
**ZDHHC13**	ZDHHC13	1.77E-03	-3.0		
**STEAP3**	STEAP3	1.92E-03	3.3	I	Copper Metabolism (16609065); Iron Metabolism (16609065); Positive Regulator of Inflammation (22689674)
**RUNX1**	RUNX1	1.94E-03	4.7	A	Positive Regulator of Osteoclast Differentiation (24606124); Positive Regulator of Treg Cell Differentiation (19917773); Th1-Associated (12835475); Transcription Factor
**TLR9**	TLR9	1.97E-03	3.4	I	Antiviral Immune Response (22344284); CD Marker; Inflammation; NFKB Signaling Pathway; Pattern Recognition Receptor; Plasmacytoid Dendritic Cell Associated (19266271); Toll-like Receptor Signaling Pathway; TIR-Containing Domain (12819136)
**UNC13D**	UNC13D	1.98E-03	-2.4	I	Positive Regulator of Inflammation (31897916); Positive Regulator of NK Cell Function (29884704); UNC13D/Munc13–4 deficiency (FHL3) (31953710)
**GAPDHS**	GAPDHS	2.03E-03	-2.9		
**SLAMF7**	SLAMF7	2.03E-03	3.9	I	CD Marker; Immunoreceptor Tyrosine-based Inhibitory Motif (ITIM) Containing (14709333); Positive Regulator of NK Cell Cytotoxicity (11698418)
**CXCL3**	CXCL3	2.03E-03	9.7	A	Asthma (23904157); CXC Chemokine; Cytokine; CXCR2 Ligand; IL-13 Induced (226114740; IL-17 Induced (28933920); Kinocidin (17208195)
**CXCL2**	CXCL2	2.03E-03	11.0	A	Asthma (15585884); CXC Chemokine; Cytokine; CXCR2 Ligand; Kinocidin (17208195)
**TNFSF9**	TNFSF9	2.05E-03	3.6	I	Apoptosis; Cytokine; Immune Checkpoint Ligand (27034234); Th1-Associated (14749528)
**CLU**	CLU	2.05E-03	5.0	A	Asthma (26826245); Complement Related; Lipid Transport (23515283); Positive Regulator of Apoptosis
**AIF1L**	AIF1L	2.46E-03	-2.5		
**PARVG**	PARVG	2.48E-03	2.4	I	Leukocyte Migration (16517730)
**CD1E**	CD1E	2.48E-03	10.0	A	CD Marker; Lipid Antigen Presentation (21844346); Dendritic Cells (h) (10948205); Langerhans Cells (h) (10948205); T Cells (h) (21844346)
**SEPT4**	ENSSSCG00000017643	2.56E-03	-2.8		
**PHACTR1**	PHACTR1	2.72E-03	4.1	A	Allergy (31838046)
**FGL2**	FGL2	2.78E-03	-2.2		Coagulation; Graft Rejection (15905589); Type 2 IFN Induced (14976252); Negative Regulator of T Cell Proliferation (18991288); Treg Associated (19085958); Secreted Immune Checkpoint Regulator (25259408)
**MCOLN2**	MCOLN2	2.80E-03	22.8	A	Chemokine Production (30479274)
**MTMR11**	MTMR11	2.91E-03	-3.0		
**YOD1**	YOD1	2.91E-03	-2.7		
**PKDCC**	PKDCC	3.07E-03	-2.1		
**MYO1F**	MYO1F	3.13E-03	2.5	A	Positive Regulator of Mast Cell Function (31143189)
**DOCK2**	DOCK2	3.13E-03	4.9	A	B Cell Chemotaxis (11518968); CDM Family; Combined immunodeficiency (CID) (31953710); Plasmacytoid Dendritic Cell Chemotaxis (18198348); Neutrophil Chemotaxis (16943182); Positive Regulator of T Cell Activation (19451552); Negative Regulator of TH2 Cell Development (17767160); T Cell Chemotaxis (11518968)
**ENPP3**	ENPP3	3.17E-03	9.5	A	Asthma (20159259); Basophil Activation Marker (15031605); CD Marker; Basophil; subset (m, h) (20159259, 23577180); Mast Cell subset (h) (12021549)
**CXCL17**	CXCL17	3.52E-03	4.4	A	Asthma (29644894) Cytokine; Dendritic Cell Chemoattractant (16455961); GPR35 Ligand (25411203)
**PIGR**	PIGR	3.52E-03	71.7	A	Allergy (19886928); Immunoglobulin Receptor (16048543); Oral Tolerance (19886928) STAT6 Induced (11034397)
**RUNX3**	RUNX3	3.55E-03	4.6	A	Negative Regulator of TH2 Development (17646405); Positive Regulator of Group 1 ILC Denevopment (ILC3) (m) (26414766); Positive Regulator of Group 3 ILC Denevolment (ILC3) (m) (26414766); Positive Regulator of Treg Cell Differentiation (19917773); Transcription Factor
**CD74**	CD74	3.65E-03	2.1	A	Antigen Processing and Presentation; CD Marker; MHC Chaperone (22306692)
**F2RL1**	F2RL1	3.65E-03	2.8	A	Asthma (15032597); Positive Regulator of Inflammation (15317291); Negative Regulator of TLR3 Signaling (19865078); Positive Regulator of TLR4 Signaling (18622013)
**HAVCR2**	HAVCR2	3.65E-03	4.6	A	Antiviral Response (25772938); Asthma (15603868); CD Marker; Galectin 9 Receptor; Inhibitory Immune Checkpoint Receptor (28258697); Negative Regulator of NK Cell Activation (h) (22383801); Negative Regulator of Transplantation Tolerance (14556006); Ferritin Receptor (16203866); Semaphorin 4A Receptor (18617884); Th2 Associated (18617884); T cell lymphoma subcutaneous panniculitis-like (31953710); T-cell immunoglobulin and Mucin Domain-containing Family; TH1-Associated (14556006)
**FCGBP**	FCGBP	3.65E-03	93.5	A	IL-13 Induced (16980555); Immunoglobulin Receptor; TFF3 Associated (21629776); Goblet Cells (24942678)
**CARD11**	CARD11	3.66E-03	6.3	A	Asthma (22075698); BCR Signaling (12867037); CARD Domain (12819136); Combined immunodeficiency (CID) (31953710); NFKB Signaling Pathway (16356856); TCR Signaling (16356856); Th1-Associated (21320717)
**UNK126***	ENSSSCG00000044295	3.75E-03	-9.7	NC	Long Non-coding RNA (LncRNA)
**SLA-DQB1**	SLA-DQB1	3.88E-03	2.2	A	Antigen Presentation; Major Histocompatibility Complex (SLA) Class II; IL-4 Induced (30974109); T Cell Co-stimulatory
**ARHGAP45**	HMHA1	3.88E-03	2.4	A	Antigen Presentation (19234124)
**SHANK2**	SHANK2	3.88E-03	55.3	A	Asthma (30792984)
**KRT18**	KRT18	3.88E-03	57.7	A	Epithelial Cell Differentiation Marker (22943429); Intermediate Filament Protein; Retinoic Acid Induced (7526151)
**CD200**	CD200	4.03E-03	2.9	A	Antiviral Response (25654642); Asthma (25569356); Negative Regulator of Basophil Function (16177086); CD Marker; CD200R Ligand (15187158); Graft Rejection (11726033); Negative Regulator of Macrophage Activation (20833375); Negative Regulator of Mast Cell Function (15557172); Vitamin D3 Induced (22334534)
**MMP25**	MMP25	4.11E-03	4.4	A	Atopy-Associated Inflammation (20337648); Metalloprotease; Eosinophils (28216055)

**SLAMF6**	SLAMF6	4.11E-03	21.6	A	CD Marker; Th1-Associated (14988414)
**LPIN2**	LPIN2	4.78E-03	-2.3		Chronic recurrent multifocal osteomyelitis and congenital dyserythropoietic anemia (Majeed syndrome) (31953710)
**IRF8**	IRF8	4.84E-03	3.7	I	Antiviral Response; B Cell Maturation Marker (15928681); Positive Regulator of Plasmacytoid Dendritic Cell Development (27637148); Resistance to T. gondii; Transcription Factor; Type 1 IFN-Induced; Type 1 IFN Signaling (15864272)
**EMILIN2**	EMILIN2	5.00E-03	-3.5		
**GIMAP1L**	GIMAP1L	5.25E-03	3.3	I	Porcine GIMAP1 paralog.
**CTXN3**	ENSSSCG00000036570	5.32E-03	-3.2		
**LY75**	LY75	5.33E-03	22.1	A	CD Marker; Cell Surface Protein; Pattern Recognition Receptor (22988114)
**WDR31**	WDR31	5.40E-03	-2.6		
**GLRX**	GLRX	5.60E-03	3.3	I	Positive Regulator of TLR4 Signaling (20539014)
**MMP7**	MMP7	5.77E-03	14.0	A	Asthma (20624652); Inflammation (15110795); IL-4 Induced (20624652); IIL-13 Induced (20624652); Metalloprotease;
**OAS2**	OAS2	5.78E-03	-5.9	I	Antiviral Response (21190483); Type 1 IFN-Induced (9790745)
**MUC20**	MUC20	6.13E-03	5.2	A	Membrane Mucin (14565953); LPS Induced (14565953)
**ITGB7**	ITGB7	6.76E-03	2.5	A	Allergy (10329831); B Cell Tolerance (22710445); Integrin; Mucosal Immunity (7687523)
**COL8A1**	COL8A1	8.14E-03	-2.7		Collagen, type VIII
**CCL5**	CCL5	8.15E-03	4.1	A	Asthma (16461122); CCL Chemokine; CCR1 Ligand; CCR3 Ligand; CCR5 Ligand; Cytokine; Kinocidin (17208195); LPS Induced (17082649)
**TIFAB**	TIFAB	8.21E-03	4.3	I	Negative Regulator of NFKB Signaling (19470519)
**GZMA**	GZMA	8.21E-03	18.2	I	Granzyme; NK Cell Cytotoxicity; Treg Associated (15485635)
**IL2RG**	IL2RG	8.22E-03	3.6	I	CD Marker; Cell Surface Protein; Cytokine Receptor; IL2 Receptor; IL4 Receptor; IL7 Receptor; IL9 Receptor; IL21 Receptor; JAK-STAT Signaling Pathway; Severe Combined immunodeficiency (SCID) (31953710)
**BIN2**	BIN2	8.41E-03	3.4	A	Endocytosis (23185384); Positive Regulator of Mast Cell Function (23185384)
**KLRK1**	KLRK1	8.41E-03	3.9	I	CD Marker; Killer Cell Lectin-like Receptor Subfamily; Positive Regulator of NK Cell Activity (18979516)
**MZB1**	MZB1	8.41E-03	18.7	A	Positive Regulator of Immunoglobulin Synthesis (21093319
**CYP1B1**	CYP1B1	8.43E-03	-2.5		Estradiol Metabolism (23821647); Xenobiotic Metabolism (27367670)
**IL2RB**	IL2RB	8.65E-03	11.1	A	Cytokine Receptor; CD122 Deficiency (31953710); CD Marker; IL2 Receptor; IL15 Receptor; Immune Checkpoint Receptor
**UNK124***	ENSSSCG00000042666	8.66E-03	-8.1	NC	Long Non-coding RNA (LncRNA)
**HOXB5**	HOXB5	8.72E-03	-5.4		Antennapedia Family; Development; Homeobox Transcription Factor
**ARHGAP4**	ARHGAP4	8.80E-03	2.7	A	Antigen Processing and Presentation (19234124)
**MYB**	MYB	8.80E-03	4.1	I	Asthma (19198610, 23606399); B Cell Differentiation (14982952); Myeloid Cell Differentiation (22918254); T Cell Differentiation (10323859); TGFB Induced (22101269); Transcription Factor
**JAK3**	JAK3	9.64E-03	3.6	I	CXCR4 Signalling; IL2 Receptor Signaling; IL4 Receptor Signaling; IL7 Receptor Signaling; IL9 Receptor Signaling; IL15 Receptor Signaling; Severe Combined immunodeficiency (SCID) (31953710); TH2-Associated (10839803); TNFRSF5 Signaling Pathway
**SLA-DRA**	SLA-DRA	9.98E-03	2.2	A	Antigen Presentation; Major Histocompatibility Complex (SLA) Class II; IL-4 Induced (30974109); T Cell Co-stimulatory
**AGR2**	AGR2	9.98E-03	66.4	A	Asthma (22403803); IL-13 Induced (21275604); Positive Regulator of Mucin Biosynthesis (24942678)
**DDX58**	DDX58	1.00E-02	-7.0	I	Antiviral Immune Response (15208624, 27318973); LPS Induced (11890704); Pattern Recognition Receptor; Retinoic Acid Induced (11890704); RIG-I-Like Receptor Family; Type 1 IFN-Induced (15208624); DExD/H-Box Helicase
**CFB**	CFB	1.00E-02	7.9	A	Factor B Deficiency (31953710); Factor B GOF (31953710); Positive Acute Phase Reactant; Alternative Complement Activation Pathway
**ZG16B**	ZG16B	1.05E-02	179.2	A	Goblet Cells (24942678)
**KCNN4**	KCNN4	1.08E-02	51.7	A	Asthma (26689552); IL-4 Induced (25071444) Positive Regulator of T Cell Activation (18796614)
**WFDC21**	WFDC21	1.09E-02	-2.8	I	Negative Regulator of Dendritic Cell Differentiation (h) (24744378); Negative Regulator on STAT3 Signalling (h) (24744378)
**LYN**	LYN	1.11E-02	3.3	A	Asthma (16034130); Negative Regulator of TLR2 Signaling (20385881); Negative Regulator of TLR4 Signaling (20385881); Positive Regulator of BCR Signaling; Positive Regulator of FCER Signaling; Positive Regulator of Dendritic Cell Function (22491248)
**CHIA**	CHIA	1.11E-02	40.9	A	Asthma (15192232); Chitinase and FIZZ Family (ChaFF); GH18 Chitinase Like Superfamily (17594485); Negative Regulator of Apoptosis (19342690); TH2-Associated (11553626); Resistance to Ascaris (27977724)
**CHRDL1**	CHRDL1	1.13E-02	-2.6		
**CD5**	CD5	1.23E-02	8.6	A	CD Marker; B Cell Surface Protein
**PTPN6**	PTPN6	1.25E-02	2.2	A	Allergy (12511594); JAK-STAT Signaling Pathway; Mast Cell Activation (10640770); Negative Regulator of BCR Signaling; Negative Regulator of TCR Signaling
**ICAM1**	ICAM1	1.25E-02	2.3	A	Adhesion Molecule (10924857); Asthma (24689994); CD Marker; Cell Surface Protein; Eosiinophilic Esophagitis (24870064); NFKB Signaling Pathway; Rhinovirus Receptor
**LRRC31**	LRRC31	1.32E-02	59.1	A	Eosinophilic Esophagitis (26462420); IL-13 Induced (26462420)
**GPR183**	GPR183	1.33E-02	6.3	A	B Cell Development (19615922); IL-4 Induced; Oxysterol Receptor (21796212)
**KCNJ11**	KCNJ11	1.34E-02	-3.1		
**S100A1**	S100A1	1.36E-02	27.4	I	Follicular Dendritic Cell Associated (16470387);
**CXCR6**	CXCR6	1.38E-02	17.1	A	CD Marker; Chemokine Receptor; CXCL16 Receptor; Cytokine Receptor; TH1-Associated (12044980); TH17 Associated
**SLC26A7**	SLC26A7	1.38E-02	65.7	A	Chloride Transporter (11834742); Oxalate Transporter (11834742); Sulfate Transporter (11834742)
**ANPEP**	ANPEP	1.41E-02	10.5	A	Aminopeptidase; Angiogenesis (12406907); Basophil Activation Marker (15916720); CD Marker; Cell Surface Protein; Eosinophil Activation Marker (12554354); PEDV Receptor (20074871); TGEV Receptor (1350661)
**IGHA1**	IGHA1	1.41E-02	61.6	I	Immunoglobulin heavy constant alpha 1 Constant Region
**MAMDC2**	MAMDC2	1.42E-02	-2.5		
**KLHL6**	KLHL6	1.42E-02	2.6	I	Positive Regulator of BCR Signaling (16166635); M2a Macrophages (23293084)
**PLXNC1**	PLXNC1	1.42E-02	3.2	I	CD Marker; Negative Regulator of Dendritic Cell Activation (15611227); Positive Regulator of Inflammation (24890788)
**NFKBIZ**	NFKBIZ	1.42E-02	4.1	I	Inflammation (11086164); Positive Regulator of NFKB Signaling (19783680); Positive Regulator of IFNG Production (19707556); Positive Regulator of IL6 Production (19783680); Toll-like Receptor Signaling Pathway (12565889)
**THBS1**	THBS1	1.42E-02	5.4	I	Adhesion Molecule; Adipocytokine (18057090); Cytokine; Negative Regulator of Angiogenesis (15522326); Negative Regulator of Phagocytosis (21573017)
**SHC2**	SHC2	1.43E-02	-2.2		VEGFR Signaling (12214271)
**GCNT4**	GCNT4	1.47E-02	-3.3		
**MX1**	MX1	1.48E-02	-8.4		Antiviral Immune Response (25572883); Type 1 IFN Induced (25572883)
**TNFSF8**	TNFSF8	1.48E-02	10.2	A	Apoptosis; B Cell Activation Marker; CD Marker; Cell Surface Protein; Graft-Versus-Host Disease (15322151); T Cell Activation Marker; TH2-Associated (11728464); TNF Superfamily
**SPDEF**	SPDEF	1.48E-02	83.4	A	Asthma (19614611); Positive Regulator of Goblet Cell Differentiation (19786015)
**NCF4**	NCF4	1.51E-02	2.5	A	Asthma (29679657); Autosomal Recessive Chronic Granulomatous Disease (CGD) (31953710); Crohn’s Disease (17435756); NADPH Oxidase Complex; IL-4 Repressed (9189052); Superoxide Generation
**C6orf15**	C6orf15	1.52E-02	-3.7		
**H2BC21**	H2BC21	1.52E-02	-2.1		
**KPRP**	ENSSSCG00000006598	1.56E-02	-4.9		Positive Regulator of Epidermal Barrier Function (30905808); , Decreased expression during atopic dermatitis (30905808)
**ISG20**	ISG20	1.56E-02	-3.4		
**SPINK5**	SPINK5	1.56E-02	-3.1		
**TRIOBP**	TRIOBP	1.59E-02	-2.9		
**MUC19**	MUC19	1.60E-02	61.9	A	IL-4 Induced IL-13 Induced; Salivary Mucin (26701274); Secreted Gel Forming Mucin
**CEP85L**	CEP85L	1.61E-02	-2.4		
**LAG3**	LAG3	1.65E-02	7.1	A	Asthma (31534137); CD Marker; Immune Checkpoint Receptor (28990586); MHC Class II Receptor; Th1-Associated (10331500); Treg-Associated (15634887)
**MUC4**	MUC4	1.68E-02	9.2	A	Membrane Mucin; IL-9 Induced (16779668); Retinoic Acid Induced (10938282); Salivary Mucin (26701274)
**TFF3**	TFF3	1.75E-02	108.0	A	Epithelial Wound Healing (16410243); IL-4 Induced (15004182); FCGBP Associated (21629776); Goblet Cells (24942678); STAT6 in promoter of human gene (15004182); SPDEF in promoter of porcine gene (29555969)
**IGHE**	IGHE	1.76E-02	27.4	A	Immunoglogulin E constant region; Allergy; Antiparasitic Response (24592267); Asthma
**SERPINB12**	SERPINB12	1.78E-02	-3.4		
**TSPEAR**	TSPEAR	1.78E-02	-2.4		
**TF**	TF	1.82E-02	-2.6	I	Iron Transport; Negative Acute Phase Reactant
**SYTL1**	SYTL1	1.82E-02	-2.1	I	Positive Regulator of Superoxide Production (11278853);
**CD53**	CD53	1.85E-02	4.7	A	Asthma (23313165); CD Marker; Cell Surface Protein; LPS Induced (15055538); Positive Regulator of Apoptosis (12606948); Positive Regulator of B Cell Activation (7963560)
**ABLIM3**	ABLIM3	1.92E-02	-2.7		
**C16orf89**	C16orf89	1.96E-02	-2.4		
**CST6**	CST6	1.96E-02	-2.3		Cysteine Protease Inhibitor
**ZNF503**	ZNF503	1.96E-02	-2.2		
**SPATS2L**	SPATS2L	1.97E-02	-1.8	A	Asthma (22792082); Psoriasis (24267790)
**MFAP5**	MFAP5	1.99E-02	-2.7	I	Extracellular Matrix Protein; Positive egulato of Neutrophil Development (23963447)
**FXYD1 /FXYD7**	ENSSSCG00000021374	2.01E-02	-2.3		

**ARL9**	ARL9	2.01E-02	-2.2		
**USP25**	USP25	2.01E-02	-1.8	I	Postive Regulator of Antiviral Response (m) (26305951); Ubiquitin-specific Protease (10644437)
**PREX1**	PREX1	2.04E-02	2.3	A	Asthma (30312708); Positive Regulator of Neutrophil Function (m) (16243036); Positive Regulator of Macrophage Function (m) (18502673)
**SIGLEC10**	SIGLEC10	2.16E-02	-2.4	I	Adhesion Molecule; CD Marker; B1 B Cell Marker (m) (17572677); CD52 Receptor (23685786); Immunoreceptor Tyrosine-based Inhibitory Motif (ITIM) Containing (12163025); PRRSV Receptor (28742001); Negative Regulator of T Cell Activation (h) (23685786)
**TNNC1**	TNNC1	2.17E-02	-5.0		
**ABCC8**	ABCC8	2.18E-02	-3.1		ATP-sensitive Potassium Channel
**ESYT3**	ESYT3	2.18E-02	-2.6	A	IL-4 Repressed (30974109)
**SMOX**	SMOX	2.30E-02	-2.5		Negative Regulator of Asthmatic Inflammation (29729200); Polyamine Metabolism
**LMO3**	ENSSSCG00000036499	2.30E-02	-3.6		
**PDCL2**	PDCL2	2.30E-02	-2.2		
**MXRA7**	ENSSSCG00000037376	2.30E-02	-2.1		Tissue Remodeling (29781547)
**LOC100737115**	ENSSSCG00000048558	2.30E-02	-2.1		Long Non-coding RNA (LncRNA)
**USP53**	USP53	2.30E-02	-2.1		
**TSC22D2**	TSC22D2	2.30E-02	-2.1		
**DSTN**	DSTN	2.30E-02	-2.1	I	Negative Regulator of Intestinal Inflammation (26878213)
**PRR13**	PRR13	2.30E-02	-2.1		
**ACTA2**	ACTA2	2.30E-02	-2.1		
**SPATA20**	SPATA20	2.30E-02	-2.1		
**KMT2B**	KMT2B	2.30E-02	-2.1		Epigenetic Regulation; H3-K4 Histone Methyltrasferase (17166833)
**RNASE1**	RNASE1	2.30E-02	-2.0		
**SMIM13**	ENSSSCG00000032146	2.30E-02	-2.0		
**MCC**	MCC	2.30E-02	-2.0		Negatively Regulator of Cell Cycle
**NABP1**	NABP1	2.30E-02	-2.0		
**C18orf25**	C18orf25	2.30E-02	-2.0		
**CAMK2G**	CAMK2G	2.30E-02	-2.0	I	Negative Regulator of TCR Signaling (16002660); Positive Regulator of STAT3 Signaling (28319059)
**SASH1**	SASH1	2.30E-02	-2.0	I	Positive Regulator of TLR4 Signaling (23776175)
**SPAG1**	SPAG1	2.30E-02	-2.0		
**ZFAND6**	ZFAND6	2.30E-02	-2.0		
**FAM20A**	FAM20A	2.30E-02	-2.0		
**VEGFD**	VEGFD	2.30E-02	-2.0	I	Cytokine
**IQSEC2**	IQSEC2	2.30E-02	-2.0		
**UNK127***	ENSSSCG00000041487	2.30E-02	-2.0		Long Non-coding RNA (LncRNA)
**DUSP3**	DUSP3	2.30E-02	-2.0	I	Negative Regulator of STAT5 Signaling (17785772); Negative Regulator of TCR Signaling (11085983)
**PLA2G4B**	PLA2G4B	2.30E-02	-2.0	I	Antibacterial Response (11741884); Neutrophils (11741884)
**TRMT9B**	TRMT9B	2.30E-02	-2.0		
**DYNLT3**	DYNLT3	2.30E-02	-1.9		
**SORBS1**	SORBS1	2.30E-02	-1.9		
**GHSR**	GHSR	2.30E-02	-1.9		Neurotransmitter Receptor
**FBN1**	FBN1	2.30E-02	-1.9		
**ITGB8**	ITGB8	2.30E-02	-1.9		
**MYL6**	ENSSSCG00000039506	2.30E-02	-1.9		
**GLB1**	GLB1	2.30E-02	-1.9		
**B4GALT5**	B4GALT5	2.30E-02	-1.9		Biosynthesis of Lactosylceramide (20574042)
**DCTN1**	DCTN1	2.30E-02	-1.9		
**UBL3**	UBL3	2.30E-02	-1.9		
**LPL**	LPL	2.30E-02	-1.8	N	Adipocyte Differentiation Marker; Cholesterol Metabolism; Lipid Metabolism; Triglyceride Metabolism; Vitamin A Metabolism (21795711)
**KCTD15**	KCTD15	2.30E-02	-1.8		
**BTD**	BTD	2.30E-02	-1.8	N	Biotin Metabolism
**INPP5A**	INPP5A	2.30E-02	-1.8		
**GPSM2**	GPSM2	2.30E-02	-1.8		
**SPSB3**	SPSB3	2.30E-02	-1.8		
**RBKS**	RBKS	2.30E-02	-1.8		
**R3HCC1L**	ENSSSCG00000010531	2.30E-02	-1.8		
**EHD4**	EHD4	2.30E-02	-1.8		Endosomal Transport (18331452)
**MIER3**	MIER3	2.30E-02	-1.8		
**ATP8B1**	ATP8B1	2.30E-02	-1.8		
**UBE2H**	UBE2H	2.30E-02	-1.7		
**PAK6**	PAK6	2.30E-02	-1.7		
**YPEL3**	YPEL3	2.30E-02	-1.7		
**HECTD1**	HECTD1	2.30E-02	-1.7		
**CAVIN2**	CAVIN2	2.30E-02	-1.7		
**NECAP2**	NECAP2	2.30E-02	-1.7		Positive Regulator of Endocytosis (27206861)
**TACC2**	TACC2	2.30E-02	-1.7		
**DAGLA**	DAGLA	2.30E-02	2.0		Endocannabinoid Metabolism (26082754); Lipid Metabolism (26731274)
**AKNA**	AKNA	2.30E-02	2.3		Negative Regulator of Inflammation (21606955); Transcription Factor
**EVA1C**	EVA1C	2.30E-02	2.3		Heparin Binding (19470522); SLIT Receptor (24040182)
**LPXN**	LPXN	2.30E-02	2.6	I	Negative Regulator of BCR Signaling (17640867); Positive Regulator of Osteoclast function (16914530)
**EPB41L4B**	EPB41L4B	2.30E-02	2.9		Wound Healing (23664528)
**KIF21B**	KIF21B	2.30E-02	2.9		
**NEK6**	NEK6	2.30E-02	3.0		
**LRRC8B**	LRRC8B	2.30E-02	3.1		
**ANKRD6**	ANKRD6	2.30E-02	3.1		
**ADAMTS3**	ADAMTS3	2.30E-02	3.3		Metalloprotease; Tissue Remodeling (26740262)
**PRR16**	PRR16	2.30E-02	3.3		
**GPR148L***	GPR148L*	2.30E-02	3.5		
**CORO1A**	CORO1A	2.30E-02	3.5		Positive Regulator of Phagocytosis (17442961); Severe Combined immunodeficiency (SCID) (31953710)
**P2RY13**	P2RY13	2.30E-02	3.6		Purinergic Receptor
**CCDC88B**	CCDC88B	2.30E-02	3.7		Endoplasmic Reticulum Stress Response (21289099)
**CADPS2**	CADPS2	2.30E-02	3.7		
**IPCEF1**	IPCEF1	2.30E-02	4.0		Oxidative Stress Response (11320098)
**GK**	GK	2.30E-02	4.8		Triglyceride Metabolism (8651297)
**KCNE3**	KCNE3	2.30E-02	4.9		Voltage-gated Potassium Channel
**ST3GAL5**	ST3GAL5	2.30E-02	5.5		Biosythesis of Ganglioside GM3
**CSMD2**	CSMD2	2.30E-02	5.6		
**RND1**	RND1	2.30E-02	6.4		
**RAC2**	RAC2	2.30E-02	6.6		Antibacterial Response (22018470); Th1-Associated (10864872); Severe Combined immunodeficiency (SCID) (31953710)
**ARSF**	ARSF	2.30E-02	6.6		Estrogen Metabolism
**SLC45A3**	SLC45A3	2.30E-02	6.9		Glucose Transporter (22521588)
**GABRP**	GABRP	2.30E-02	7.4		GABA Receptor; Neurotransmitter Receptor
**C2CD4B**	C2CD4B	2.30E-02	7.8		IL-1b induced (15527968)
**SLC6A14**	SLC6A14	2.30E-02	8.2		Beta alanine Transporter (18599538)
**PLEKHD1**	PLEKHD1	2.30E-02	8.5		
**RASSF5**	RASSF5	2.30E-02	8.7		
**CYTIP**	CYTIP	2.30E-02	8.8	I	Negative Regulator of Dendritic Cell Function (22488362)
**MYO5C**	MYO5C	2.30E-02	8.8		Myosin
**ACSS3**	ACSS3	2.30E-02	9.5	N	Lipid Metabolism (28003429); Short-chain ACS Family (17762044)
**PKIB**	PKIB	2.30E-02	10.0		
**PIP5K1B**	PIP5K1B	2.30E-02	10.1		
**EDAR**	EDAR	2.30E-02	10.3		Cytokine Receptor
**TSPAN1**	TSPAN1	2.30E-02	10.6		Thiamine Transport (21836059)

**PGLYRP1**	PGLYRP1	2.30E-02	10.6	I	Pattern Recognition Receptor (15956276)
**IRX2**	IRX2	2.30E-02	10.8		Development; TALE (Three Amino Acid Loop Extension) Homeobox Family Transcription Factor
**RIPOR2**	RIPOR2	2.30E-02	11.3		Positive Regulator of T Cell Migration (23241886); Positive Regulator of T Cell Proliferation (30254631, 27556504)
**FRK**	FRK	2.30E-02	11.7		
**FAM184B**	FAM184B	2.30E-02	12.1		
**HAL**	HAL	2.30E-02	12.5		Amino Acid Catabolism
**SLC28A3**	SLC28A3	2.30E-02	12.8		Nucleoside Transporter (15667311)
**KLHDC8A**	KLHDC8A	2.30E-02	13.3		
**SEL1L3**	SEL1L3	2.30E-02	13.4		
**TNIP3**	TNIP3	2.30E-02	13.5	I	Adiponectin Induced (19617629); IL10 Induced (17485448); LPS Induced (17088249); Negative Regulator of NFkB Signaling (17088249); Negative Regulator of TLR4 Signaling (17088249)
**SOX9**	SOX9	2.30E-02	13.6		Development; Positive Regulator of Paneth Cell Differentiation (17698607); Transcription Factor
**MATN1**	MATN1	2.30E-02	13.9		
**ADAMTS18**	ADAMTS18	2.30E-02	14.0		Metalloprotease
**RFLNA**	ENSSSCG00000035760	2.30E-02	14.5		
**TMEM213**	TMEM213	2.30E-02	14.6		
**RGS1**	RGS1	2.30E-02	15.5		Negative Regulator of CXCR4 Signaling (15728464)
**SLC16A10**	SLC16A10	2.30E-02	15.9	N	Aromatic Amino Acid Transporter; Phenylalanine Transporter (23045339); Tryptophan Transporter (23045339); Tyrosine Transporter (23045339)
**PI3**	PI3	2.30E-02	16.0		Antimicrobial Peptide Activity (17964057)
**COL9A2**	COL9A2	2.30E-02	16.1		Collagen, type IX
**ALX4**	ALX4	2.30E-02	17.1		PRD Class Homeobox Transcription Factor
**TESC**	TESC	2.30E-02	17.2	I	Positive Regulator of Megakaryocytic Differentiation (17717601)
**TFCP2L1**	TFCP2L1	2.30E-02	17.4		Negative Regulator of Cell Cycle
**SLCO4A1**	SLCO4A1	2.30E-02	17.6		Steroid Sulfate Transporter
**PAH**	PAH	2.30E-02	18.4	N	Amino Acid Metabolism; Phenylalanine Catabolism; Tyrosine Biosynthesis
**F5**	F5	2.30E-02	20.9		Coagulation (12393635)
**SLC17A9**	SLC17A9	2.30E-02	21.6		Nucleotide Transporter
**STAC2**	STAC2	2.30E-02	22.9		
**BFSP1**	BFSP1	2.30E-02	23.0		Intermediate Filament Protein
**SCGN**	SCGN	2.30E-02	23.4		
**TMC5**	TMC5	2.30E-02	23.7		
**UNK125***	ENSSSCG00000049705	2.30E-02	24.1	NC	Long Non-coding RNA (LncRNA)
**SIX2**	SIX2	2.30E-02	24.1		Development; Homeobox Transcription Factor
**RFX6**	RFX6	2.30E-02	25.9		Daiabetes (21965172)
**TCEANC2L***	ENSSSCG00000045593	2.30E-02	26.1	NC	Long Non-coding RNA (LncRNA)
**SIDT1**	SIDT1	2.30E-02	26.9		Cholesterol Transport (28785058); dsRNA Transport
**PLEKHS1**	PLEKHS1	2.30E-02	27.3		
**GPRC6A**	GPRC6A	2.30E-02	27.9		L-Arginine Receptor (24032653); L-lysine Receptor (24032653); L-Ornithine Receptor (24032653);
**F7**	F7	2.30E-02	30.8		Vitamin K-dependent Coagulation Factor
**LAMA1**	LAMA1	2.30E-02	31.0		Extracellular Matrix Glycoprotein
**CRACR2A**	CRACR2A	2.30E-02	31.1		Ca2+Release-activated Ca2+ Channel; Positive Regulator of T Cell Activation (20418871)
**NKX3-1**	NKX3-1	2.30E-02	31.2		Antennapedia Family; Development; NKL Subclass Homeobox Transcription Factor
**UNK96***	ENSSSCG00000050015	2.30E-02	32.0	NC	Long Non-coding RNA (LncRNA)
**WAP1***	WAP1	2.30E-02	32.1		Porcine-specific gene.
**FOXI1**	FOXI1	2.30E-02	34.0		Forkhead Transcription Factor
**KRT8**	KRT8	2.30E-02	34.4		Type II Keratin Family
**CLDN3**	CLDN3	2.30E-02	35.1		Adhesion Molecule; Bacterial Enterotoxin Receptor (9334247); Terminal Mediator of Cytokine-induced Regulation of Intestinal Tight Junction Paracellular Permeability (18952050); Tight Junction Associated (12909588)
**SLC5A5**	SLC5A5	2.30E-02	36.2		Iodide Tranporter
**SLC5A8**	SLC5A8	2.30E-02	36.7		Iodide Transporter
**SLC16A11**	SLC16A11	2.30E-02	38.2		
**FAM177B**	FAM177B	2.30E-02	38.3		
**SERPINA1**	SERPINA1	2.30E-02	39.2		Positive Acute Phase Reactant; Serine Protease Inhibitor; M2c Macrophages (28318799); Vitamin D3 Induced (30690074)
**CHRM1**	CHRM1	2.30E-02	40.7		Cholinergic Receptor; Muscarinic Receptor; Neurotransmitter Receptor
**IRX1**	IRX1	2.30E-02	40.7		Development; TALE (Three Amino Acid Loop Extension) Homeobox Family Transcription Factor
**ZBED2**	ZBED2	2.30E-02	41.2	I	Negative Regulator of IRF1 Signaling (32385160)
**POU2AF1**	POU2AF1	2.30E-02	41.6		B Cell Differentiation (12857960); Th1 Associated (17568779); Transcriptional Co-activator
**IGLL1L2***	ENSSSCG00000041880	2.30E-02	42.9		
**BPIFB6**	BPIFB6	2.30E-02	44.8	I	Antiviral Response (26962226; Bacterial Binding (12185532)
**SLC9A4**	SLC9A4	2.30E-02	47.5		Intracellular pH Regulator (22049213)
**OLFM4**	OLFM4	2.30E-02	47.6		Negative Regulator of Antibacterial Responses (20534456, 22844115); Negative Regulator of NFKB Activation (20534456)
**CLGN**	CLGN	2.30E-02	48.8		
**KIAA1324**	KIAA1324	2.30E-02	49.2		Autophagy (21072319)
**IRX4**	IRX4	2.30E-02	50.1		Development; TALE (Three Amino Acid Loop Extension) Homeobox Family Transcription Factor
**ABCC4L5***	ABCC4L5*	2.30E-02	51.3		Porcine paralog.
**WFDC2**	WFDC2	2.30E-02	51.4		Cystic Fibrosis (27105680); Proteiase Inhibitor (23139753);
**LILRB3L2***	LILRB3L2	2.30E-02	63.5		
**CLDN8**	CLDN8	2.30E-02	72.3		Claudin Family; Negative Regulator of Paraceluular Na+ Transport (19000657)
**ASGR2**	ASGR2	2.30E-02	78.4		Pattern Recognition Receptor (16286643)
**HPD**	HPD	2.30E-02	88.5		Amino Acid Metabolism; Phenylalanine Catabolism
**CYP2B22**	CYP2B22	2.30E-02	89.9		
**RNF182**	RNF182	2.30E-02	100.4		LPS Induced (31432514); Negative Regulator of TLR Signaling (31432514); Zn Containing
**UNK123***	ENSSSCG00000046121	2.30E-02	113.5	NC	Long Non-coding RNA (LncRNA)
**CYP4A11**	ENSSSCG00000003754	2.30E-02	114.4		
**LOC106506585**	ENSSSCG00000048583	2.30E-02	160.4		Long Non-coding RNA (LncRNA)
**PPP1R1B**	PPP1R1B	2.30E-02	571.8		
**OBP2B**	OBP2B	2.30E-02	1052.9		
**KCTD4**	KCTD4	2.31E-02	-4.5		
**MXRA8**	MXRA8	2.31E-02	-2.3		
**PHLDA1**	PHLDA1	2.43E-02	3.0	A	Asthma (29977241); Positive Regulator of Apoptosis (8673705); Homocysteine Induced (12738777); IL-4 Induced (p) (30974109)
**SPARCL1**	ENSSSCG00000004212	2.46E-02	-2.7	A	Asthma (31195152)
**FCAMR**	ENSSSCG00000030821	2.46E-02	10.7	A	CD Marker; Immunoglobulin A Receptor; Immunoglobulin M Receptor;
**BATF3**	BATF3	2.48E-02	4.2	A	Positive Regulator of Dendritic Cell Development (20351058); Posiive Regulator of IL-9 Synthesis (31776325); Resistance to T. gondii Infection (21867928); Transcription Factor
**RNF222**	RNF222	2.50E-02	-3.3		
**BARX2**	BARX2	2.53E-02	-2.3		Antennapedia Family; Development; NKL Subclass Homeobox Transcription Factor
**PPP1R12B**	PPP1R12B	2.56E-02	-2.3		
**DHX58**	DHX58	2.71E-02	-6.6	I	Antiviral Immune Response (22344284); Pattern Recognition Receptor (16116171); Negative Regulator of DDX58 Function (16210631); Negative Regulator of IFIH1 Function (16210631); RIG-I-Like Receptor Family
**FLT3**	FLT3	2.74E-02	4.1	A	B Cell Maturation Marker (15928681); Class III Receptor Tyrosine Kinase Family (RTK); Cytokine Receptor; CD Marker; Cell Surface Protein; Negative Regulator of Allergic Inflammation (19348927)
**DDX59**	DDX59	2.75E-02	-2.7		
**GPR65**	GPR65	2.77E-02	3.8	I	Inflammatory Bowel Disease (30535144, 30616622); Negative Regulator of Inflammation (21238451, 22074830); Positive Regulator of Apoptosis (15485889)
**TAS2R41**	TAS2R41	2.78E-02	-4.2		
**GALR1**	GALR1	2.81E-02	-3.6		
**SLC12A2**	SLC12A2	2.93E-02	7.2	A	Asthma (11544191); IL-4 Induced (277862590; Sodium/Chloride Cotransporter
**TLR2**	TLR2	3.05E-02	9.8	A	Asthma (14978071); CD Marker; Inflammation; NFKB Signaling Pathway; Pattern Recognition Receptor; Toll-like Receptor Signaling Pathway
**DEGS2**	DHRS9	3.05E-02	-2.3	A	Retinol Dehydrogenase (11304534); Vitamin A Metabolism (11304534); Retinoic Acid Induced (11304534)
**NRG2**	NRG2	3.05E-02	2.7	I	Growth Factor; ERBB3 Ligand; ERBB4 Ligand; EGF Family
**SAA2**	SAA2	3.09E-02	8.2	A	Positive Acute Phase Reactant

**GABARAPL2**	GABARAPL2	3.14E-02	-1.7	I	Resistance to Toxoplasma gondii (28604719, 32094251); Type 2 IFN Induced (28604719)
**A2ML1**	A2ML1	3.20E-02	-3.3		
**ANGPTL1**	ANGPTL1	3.21E-02	-2.6		Angiopoietin Family; Positive Regulator of Angiogenesis (16685428); Vascular Endothelial Growth Factor (VEGF) Family
**MILR1**	MILR1	3.21E-02	3.2	A	Allergy (25007884); Negative Regulator of Allergic Responses (31900344); Negative Regulator of Mast Cell Activation (20526344)
**ZRANB1**	ZRANB1	3.25E-02	-1.5	I	TRAF6 Binding (11463333)
**ITK**	ITK	3.32E-02	7.2	A	Combined immunodeficiency (CID) (31953710); Positive Regulator of CD28 Signaling; Positive Regulator of IL17A production (19818650); Positive Regulator of TCR Signaling (15803148); TEC Kinase Family (15803148); Th2-Associated (15345221)
**SLC26A4**	SLC26A4	3.32E-02	81.9	A	Asthma (23314903); Chloride Anion Exchanger; IL-4 Induced (28365704); IL-13 Induced (28378089); Thiocyanate Transport (27864363)
**SLA-DRB2**	SLA-DRB2	3.37E-02	2.1	A	Antigen Presentation; Major Histocompatibility Complex (SLA) Class II; IL-4 Induced (30974109); T Cell Co-stimulatory
**SASH3**	SASH3	3.37E-02	3.2	I	Lymphocyte signaling adaptor protein (11470164); Positive Regulator of B Cell Development (m) (18950867); Positive Regulator of T Cell Development (m) (19604361)
**ACP5**	ACP5	3.37E-02	4.5	A	IL-4 Induced (19801646); Iron Transporter (3527760); Spondyloenchondro-dysplasia with Immune Dysregulation (SPENCD) (31953710)
**TAC4**	TAC4	3.37E-02	37.5	A	Positive Regulation of IgE-mediated Mast Cell Function (25201259); Ulcerative Colitis (21342363)
**ISG15**	ISG15	3.37E-02	-9.9	I	Antiviral Immune Response (24448099, 28869005); Type 1 IFN-Induced (16009940)
**ERN2**	ENSSSCG00000040637	3.37E-02	48.4	A	Positive Regulator of Mucin Production (23168839)
**RNF183**	RNF183	3.37E-02	-2.4		Inflammatory Bowel Disease (26818663)
**RUNX2**	RUNX2	3.40E-02	4.1	A	Asthma (27825108); IL-4 Induced (30974109); IL-13 Induced (31331833); Positive Regulator of Goblets Cell Differentiation (27825108); Positive Regulator of Osteoblast Differentiation (30987410); Transcription Factor
**SLA-DRB1**	SLA-DRB1	3.40E-02	2.6	A	Antigen Presentation; Major Histocompatibility Complex (SLA) Class II; IL-4 Induced (30974109); T Cell Co-stimulatory; Vitamin D Regulated (19197344)
**CD3E**	CD3E	3.40E-02	6.3	A	CD Marker; Cell Surface Protein; Inflammation; Positive Regulator of TCR Signaling; Severe Combined immunodeficiency (SCID) (31953710)
**ETV4**	ETV4	3.42E-02	8.7	A	Asthma (31506535); Transcription Factor
**ANTXR2**	ANTXR2	3.46E-02	-2.3		Pattern Recognition Receptor (21075356)
**CLCA1**	CLCA1	3.47E-02	75.7	A	Asthma (11694454, 17898169); Chloride Ion Transport (p) (11015605); IL-4 Induced (p) (32431691); IL-13 Induced (17898169); Metalloprotease (22080371)
**PLA2G15**	PLA2G15	3.74E-02	-2.4		Endogenous Lipid Antigen Processing (234935500); Vitamin D3 Repressed (31707382)
**HAND2**	HAND2	3.74E-02	-4.3		
**SMPD3**	SMPD3	3.77E-02	-4.0		
**CD69**	CD69	3.79E-02	6.8	A	Allergy (28658550); CD Marker; Cell Activation Marker; Positive Regulator of Inflammation (16983725)
**VAV1**	VAV1	3.89E-02	4.7	A	Asthma (18395227); BCR Signaling (11376343); CD28 Signaling; Guanine Nucleotide Exchange Factors (GEF); FCER Signaling; Positive Regulator of Enterocyte Differentiation (19139088); TCR Signaling (14757747); TH2 Associated (15845902)
**DEGS2**	DEGS2	3.90E-02	-2.3		Biosynthesis of Phytosphingolipids
**INKA2**	INKA2	3.90E-02	-2.2		
**SPNS3**	SPNS3	3.95E-02	-2.8		
**SLC9A2**	SLC9A2	4.02E-02	18.9	A	Positive Regulator of Intestinal Barrier Function (18719001); Sodium/Hydrogen Ion Transporter (26350456)
**IGJ**	IGJ	4.08E-02	20.7	A	Immunoglobulin J Polypeptide
**POLB**	POLB	4.10E-02	-2.5		DNA Polymerase
**ADA**	ADA	4.14E-02	-2.7	A	Adenosine Metabolism; Asthma (15032597); Obesity; TH2-Associated (12897202); Severe Combined immunodeficiency (SCID) (31953710)
**UNK128***	ENSSSCG00000043044	4.14E-02	-2.2		Long Non-coding RNA (LncRNA)
**FTH1**	ENSSSCG00000014540	4.14E-02	4.0	I	Iron Metabolism; Positive Acute Phase Reactant
**BPIFB2**	BPIFB2	4.22E-02	82.8	I	Bacterial Binding (12185532)
**AKR1C2**	AKR1C2	4.32E-02	6.6	A	Asthma (15170023); Prostaglandin D2 Biosynthesis (18508192); Prostaglandin E2 Biosynthesis (18390687)
**SELE**	SELE	4.32E-02	7.8	A	Adhesion Molecule; Asthma (12165540); CD Marker; Cell Surface Protein; Hypertension (17236131, 21881522); NFKB Signaling Pathway; TNF Induced (9006914); Xenograft Rejection (7526854)
**ST6GAL1**	ST6GAL1	4.33E-02	10.4	A	Asthma (30730306); Apoptosis (12499376); IL-13 Induced (30730306); Mucin Generation (30730306); Sialyltransferase
**MUC16**	ENSSSCG00000013714	4.36E-02	72.6	A	Membrane Mucin
**DPP4**	DPP4	4.42E-02	5.1	A	Aminopeptidase; Asthma (24303167); CD Marker; Cell Surface Protein; CXCL11 Processing; Inflammation (11589380); Serine Protease; T Cell Activation; T Cell Activation Marker (1352530); Th1-Associated (9314347)
**RNF125**	RNF125	4.60E-02	3.9	I	Antiviral Immune Response (21147464); Positive Regulator of T Cell Activation (15843525); Negative Regulator of DDX58 Function (17460044); Negative Regulator of IFIH1 Function (17460044); Negative Regulator of MAVS Function (17460044); Zn Containing
**CD44**	CD44	4.61E-02	6.7	A	Asthma (21842125, 28660189); CD Marker; Cell Surface Protein; IL-13 Induced (21842125)
**VPS45**	VPS45	4.69E-02	-1.9	I	Severe congential neutropenia 5 (SCN5)
**EVI2B**	EVI2B	4.69E-02	3.1	I	CD Marker; EVI2 Family; Positive Regulator of Myeloid Development (28186500); Type I Transmembrane Protein
**ICOS**	ICOS	4.69E-02	22.8	A	Asthma (14612664); CD Marker; Combined immunodeficiency (CID) (31953710); Immune Checkpoint Receptor; Positive Regulator of TCR Signaling; TFH Cell Associated (19380638); TH2-Associated (19888475); Treg Associated (18513999)
**BHMT2**	BHMT2	4.76E-02	43.9	N	Homocysteine Metabolism; Methionine Metabolism
**SFRP2**	SFRP2	4.84E-02	-3.9		
**FGD3**	FGD3	4.84E-02	-2.6		
**TCEAL1**	TCEAL1	4.84E-02	-2.2		
**HERC5**	HERC5	4.94E-02	-5.5	I	Antiviral Immune Response (20385878); Cell Cycle
**GCNT3**	GCNT3	4.94E-02	6.0	A	IL-13 Induced (17303715); Mucin Biosynthesis (20816165); Retinoic Acid Induced (17303715)
**SERPINB11**	SERPINB11	4.97E-02	-2.7		Serine Protease Inhibitor
**MUC5B**	ENSSSCG00000033452	4.97E-02	121.2	A	Asthma (19614611, 24317696); IL13 Induced (30431351); Retinoic Acid Induced (17646388); Salivary Mucin (26701274); Secreted Gel Forming Mucin

* A = Allergy; I = Infectious disease; N = nutritional; NC = non-coding
