# Supplementary figures and images for: Immunologic and pathologic characterization of a novel swine biomedical research model for eosinophilic esophagitis
Source: Front Allergy. 2022 Nov 14;3:1029184. doi: 10.3389/falgy.2022.1029184 (PMC9701751; doi:10.3389/falgy.2022.1029184)

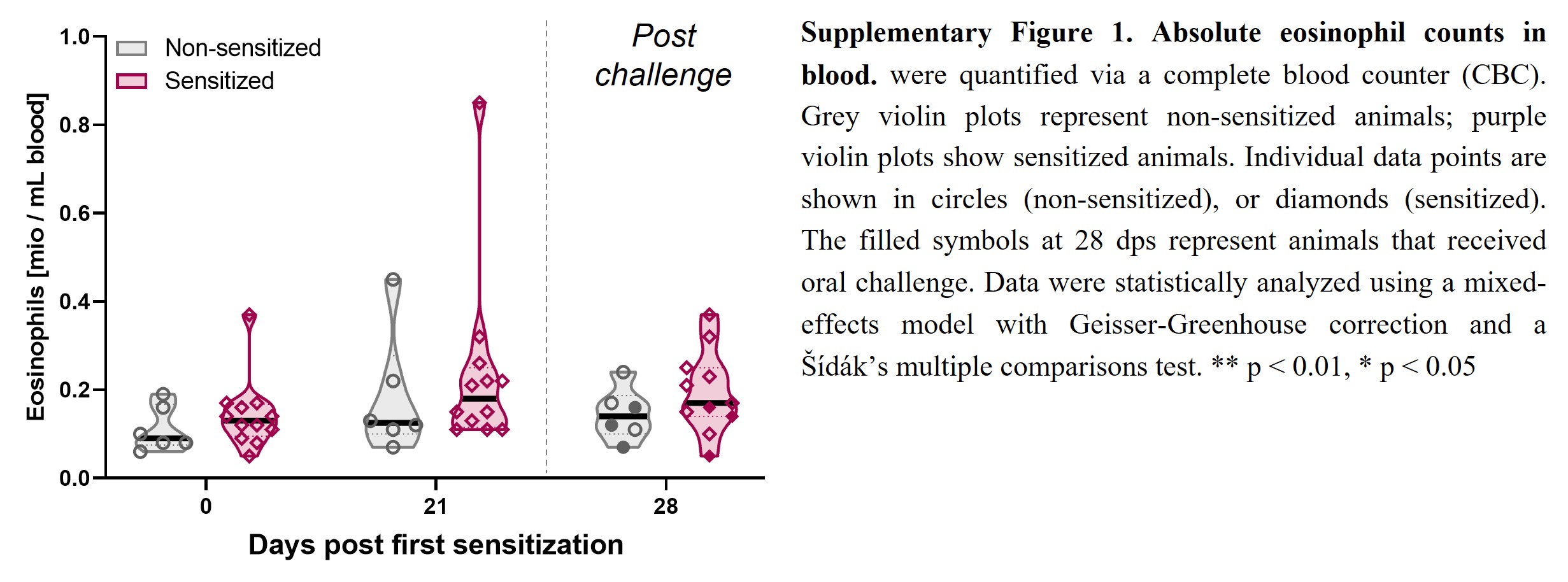

Supplement: Supplementary file 2 [file Image1.jpg]
